# A molecular view of amyotrophic lateral sclerosis through the lens of interaction network modules

**DOI:** 10.1371/journal.pone.0268159

**Published:** 2022-05-16

**Authors:** Klaus Højgaard Jensen, Anna Katharina Stalder, Rasmus Wernersson, Tim-Christoph Roloff-Handschin, Daniel Hvidberg Hansen, Peter M. A. Groenen

**Affiliations:** 1 Intomics A/S, Copenhagen, Denmark; 2 University Hospital Basel, Basel, Switzerland; 3 DTU Health Technology, Technical University of Denmark, Lyngby, Denmark; 4 Idorsia Pharmaceuticals Ltd., Allschwil, Switzerland; University of Malta, MALTA

## Abstract

**Background:**

Despite the discovery of familial cases with mutations in Cu/Zn-superoxide dismutase (SOD1), Guanine nucleotide exchange C9orf72, TAR DNA-binding protein 43 (TARDBP) and RNA-binding protein FUS as well as a number of other genes linked to Amyotrophic Lateral Sclerosis (ALS), the etiology and molecular pathogenesis of this devastating disease is still not understood. As proteins do not act alone, conducting an analysis of ALS at the system level may provide new insights into the molecular biology of ALS and put it into relationship to other neurological diseases.

**Methods:**

A set of ALS-associated genes/proteins were collected from publicly available databases and text mining of scientific literature. We used these as seed proteins to build protein-protein interaction (PPI) networks serving as a scaffold for further analyses. From the collection of networks, a set of core modules enriched in seed proteins were identified. The molecular biology of the core modules was investigated, as were their associations to other diseases. To assess the core modules’ ability to describe unknown or less well-studied ALS biology, they were queried for proteins more recently associated to ALS and not involved in the primary analysis.

**Results:**

We describe a set of 26 ALS core modules enriched in ALS-associated proteins. We show that these ALS core modules not only capture most of the current knowledge about ALS, but they also allow us to suggest biological interdependencies. In addition, new associations of ALS networks with other neurodegenerative diseases, e.g. Alzheimer’s, Huntington’s and Parkinson’s disease were found. A follow-up analysis of 140 ALS-associated proteins identified since 2014 reveals a significant overrepresentation of new ALS proteins in these 26 disease modules.

**Conclusions:**

Using protein-protein interaction networks offers a relevant approach for broadening the understanding of the biological context of known ALS-associated genes. Using a bottom-up approach for the analysis of protein-protein interaction networks is a useful method to avoid bias caused by over-connected proteins. Our ALS-enriched modules cover most known biological functions associated with ALS. The presence of recently identified ALS-associated proteins in the core modules highlights the potential for using these as a scaffold for identification of novel ALS disease mechanisms.

## Background

Amyotrophic lateral sclerosis (ALS) is a neurodegenerative disease clinically driven by deterioration of motor functions and affects about 1–2 individuals per 100,000 per year. It is a rapidly progressing disease with a life expectancy after diagnosis of 3–5 years. ALS is classified into two categories, which are clinically very similar. The most common form of ALS, with over 90% of the cases, is the sporadic form (SALS), where no mutations can be identified. The less frequent (10%) familial forms (FALS) are often autosomal dominant, caused by a number of mutations in a heterogeneous set of genes [1, 2].

The main mutations currently described are in SOD1 (20% of FALS), C9orf72 (30–40% of FALS), TARDBP and FUS (together 5% of FALS) [3]. There are also other genes that have been associated with ALS, but they represent a small proportion of cases [4]. However, there is an increasing rate of new discoveries of ALS-associated genes, for which the supporting biology is not well established. During the last 5 years, more than 7 new mutations have been identified which are associated with one or more pathological mechanisms associated with ALS [5] (MATR3, CHCHD10, TBK1, TUBA4A, NEK1, C21orf2, CCNF). These pathological mechanisms include, among others, dysfunction in global protein homoeostasis resulting from abnormal protein aggregation or a defect in the protein clearance pathway, mitochondrial dysfunction, altered RNA metabolism, impaired cytoskeletal integrity, altered axonal transport dynamics, and DNA damage accumulation due to defective DNA repair. However, despite active research, the pathogenesis and etiology of ALS remain largely unknown, and while an increasing number of genetic factors are being identified it remains unclear how these cellular events lead to the wide range of proposed mechanisms that underlie this complex and devastating neurodegenerative disease.

The clinical manifestation of ALS is not the consequence of a single mutated protein, but rather represents a complex imbalance of a dynamic network of proteins which links intra-cellular processes to inter-cellular processes. Network biology offers an approach to explore the molecular complexity of a disease and define disease modules and relevant pathways. With this approach ALS-associated mutations can be investigated in the relevant biological context of the molecular pathways they are involved in, thereby linking them to potentially new genes that may become future drug targets.

The concept of spectrum disorders has been introduced to link phenomenologically distinct diseases based on a related underlying etiology or genetic link. The most accepted spectrum disorders are in the autism spectrum, where this concept is well accepted. Also, other mental diseases, such Lewy body dementias and Parkinson’s disease are thought to be part of a spectrum rather than separate pathologies [6]. Similarly, ALS and frontotemporal dementia [7] or other motor neuron diseases have been discussed to be the extreme ends of a spectrum, based on imaging similarities and genetic and neuropathological findings [8]. Based on the ALS core modules we are analyzing other neurological disorders for molecular overlaps and are relating them to the molecular biology of ALS.

### ALS in the network biology perspective

Genes and proteins do not work in isolation, and insight about disease biology of complex diseases can be gained from investigating the interaction partners of known disease associated genes/proteins [9]. This could for example be by investigating which pathways appears to be perturbed (either by containing known disease associated proteins or by analysis of experimental data such as GWAS and gene expression studies). Pathway data from sources such as KEGG [10], WikiPathways [11] and Reactome [12] is generally of high quality, and is indeed a very valuable resource for biological interpretation of data. However, it is important to realize that pathways represent what could be considered “text-book knowledge” or “established knowledge”–a curated and aggregated summary of what is considered to be the consensus in the scientific literature. Consequently, truly novel interactions of ALS-associated genes/proteins are not expected to be found relying solely on pathway information.

Protein-protein interaction (PPI) networks can be used as an alternative avenue for analysis. PPI networks have a higher coverage of human genes/proteins compared to pathways (up to 85% coverage of proteome [13, 14]) and are especially strong in their ability to cover the areas in-between the well-known pathways because they are based on high-throughput experimental screens, rather than curation of literature knowledge. Biological modules found from network analysis of PPI data could be considered "proto-pathways" that may mature into established pathways after experimental follow-up.

When working with PPI data, it is important to be aware of experimental artifacts that may carry over into the protein networks, and which therefore may impact the interpretation of results. We explore this in detail in the supplement, as there are many factors to consider. Here we would like to emphasize two areas of concern: 1) Due to the high false-positive rate associated with PPI data, it is critically important to use a PPI resource where the experimental evidence behind each interaction has been evaluated (and where a high-confidence subset can be extracted) and 2) To appreciate that certain proteins have an inflated number of interactions in PPI networks (even after the filtering of low-confidence interactions), either due to experimental artefacts (see [15] for a review) or due to study bias. We here refer to these as over-connected proteins. This situation can adversely affect results from network analysis if not addressed properly. A protein that is highly connected due to these factors in the global interactome, will have a very high likelihood of appearing in the network neighborhood around any set of disease associated proteins, no matter if the study is investigating ALS, Heart disease, Diabetes, Cancer or any other class of disease. It will also be very likely, that such an “over-connected” protein will have a high number of interaction partners in the local network around the disease proteins being studied, without it necessarily being of particular importance for the disease *per se*. This is important, since in network analysis studies that are built around the concept of the "*centrality-lethality rule*" [16] where the node degree (number of interaction partners) is important, are likely to pick up these over-connected proteins, unless a node-degree normalization scheme is used. UBC (Polyubiquitin-C) is a good example of such an artificially over-connected protein (see Supplement for details: S3 File and S5 Table within), and we notice that this very protein is singled out as being important for ALS in recent study by Mao et al. [17], due to its high node degree in the local network around ALS associated proteins. In that study UBC’s high node degree in the entire interactome was not taken into account, and we speculate it to be a false positive.

Here, we present an approach to network analysis of known ALS-associated genes/proteins that handles the issues concerning over-connected proteins in the network in several ways. First of all, the network modules are built using a bottom-up approach, where the first-order networks around each individual ALS-associated protein are first extracted and then merged if they overlap. By doing this, rather than having a first step, where we pull out a very large network surrounding all ALS proteins, we avoid having to run a topological network clustering algorithm (such as MCODE [18] or CLUSTERONE [19]) which are prone to emphasize features in the network influenced by the over-connected proteins. As a second measure we have built in a pruning step following the initial network extraction where proteins are removed if their global degree (total number of interactions) is much larger than their local degree (number of interactions in the network being investigated), which again will down-weigh the influence of proteins that are highly and unspecifically connected in the global interactome. The result is a set of 26 ALS core modules that capture most of the current knowledge about ALS and provide new insight into the biology and etiology of this complex disease.

## Methods

**Fig 1:**
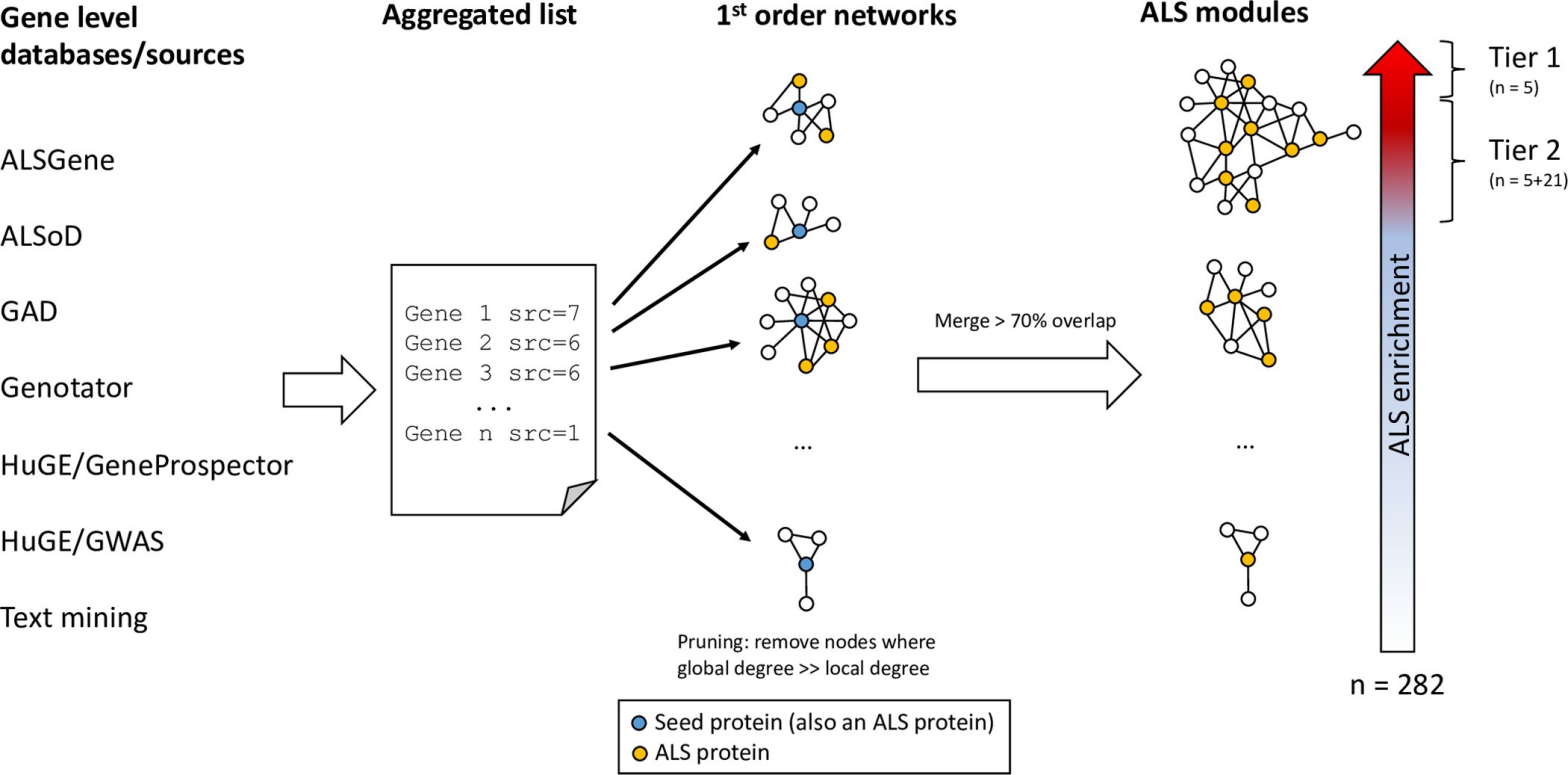
Analysis workflow. Outline of the workflow followed in this study. Briefly, lists of genes associated to ALS were collected from 7 data sources, and combined into an aggregated list, keeping note of how many sources supported each gene. Next, genes were mapped to their corresponding protein products, and for each protein, a protein-protein interaction (PPI) network of 1^st^ order interaction partners was found using the inBio Map PPI database. Networks with a large overlap were merged, producing a final set of 282 “ALS modules”. Finally, the strength of the association to ALS for each module was investigated via over-representation analysis for enrichment of ALS associated proteins. From the over-representation analysis we defined 26 modules to be “Core modules” (subdivided into two tiers of significance).

Our analysis strategy is shown in Fig 1 and is based on the concept of finding network modules of moderate size significantly enriched in known ALS-associated proteins, and where other proteins in the modules therefore are likely also involved in ALS-related processes. This also allows for a detailed analysis of the biology represented in each module, and for generating a module collection that can be used for further study.

### Overview of methods

In order to make the following sections easier to read, we first provide a brief non-technical overview of the methods:

**Step 1:** Compiling the list of known ALS-associated genes.

We compile a set of genes across multiple data sets and databases used as seed genes for PPI network building. The gene list is by purpose as inclusive and comprehensive as possible since a filtering criterion is introduced upon network generation. The gene list is generated by searching through databases of ALS-associated genes and by text mining of the scientific literature. Furthermore, we collect a set of genes that were added to the databases since the original data extraction. This gene set is then used as a benchmark set to evaluate if the ALS networks have predictive power to find (new) ALS associated genes.

**Step 2:** Generating ALS-associated PPI networks

This is the single most important step of the analysis. As reviewed extensively in both the main text and in the supplement, there are a number of potential artefacts of PPI data that needs to be addressed in the network analysis. The workflow used here (see Fig 1) is built to address these challenges (see Methods and Discussion). We use the entire gene list identified in step 1 above as seed proteins to build the PPI networks from the bottom-up. Afterwards, highly overlapping networks are merged to form the ALS modules. This workflow ensures that, even if it is impossible to completely eliminate promiscuous interactions, they will not be the drivers of the identification of the ALS modules.

**Step 3:** Defining ALS core modules

At this point, the ALS modules will each contain at least one seed protein, and many will contain multiple seed proteins. We consider the modules with multiple seed proteins (which we know have some level of support for being ALS associated), as the most interesting to investigate further. The statistical significance of the number of seed proteins in each module is assessed using Fisher’s Exact test, and we define two levels of high confidence modules (tier 1 and tier 2). By using this approach, we limit the impact of noise in the gene list from step 1, as false positives on the list will represent a random selection of genes, which are unlikely to be found in the same network, and therefore will not become significant in the statistical test.

**Step 4:** Biological annotation of the ALS network collection

The purpose of this step is to assess the biological function of each ALS module. Here, we utilize a standard Gene Ontology (GO) overrepresentation analysis as the first step, followed up by visualization of biological processes found to be significantly associated with the modules. Furthermore, a second overrepresentation analysis investigating disease associated proteins in the modules is conducted and visualized. The data set of disease associated proteins is generated using text mining (see step 1). Finally, the ALS core modules are manually inspected by ALS experts in our team for further interpretation, and selected modules are presented in details, and discussed in details in the results section.

**Step 5:** Visualization of networks

All networks and associated data are visualized using the open source program “Cytoscape”. Notice, that we provide data bundles of pre-formatted / visualized networks in the form of Cytoscape “session files” for download as part of the Supplementary Material. That allows for visual inspection of the entire collection of networks without requiring in-depth knowledge of the Cytoscape program.

### Compiling the list of known ALS-associated genes

The dataset of known ALS-associated genes, was generated by combining information from the sources listed below. The lists were selected in order to be as comprehensive and inclusive as possible in order to capture all reported ALS genes, and to allow for assessing the combined evidence for each gene (genes supported by a few data sources would be considered less certain, compared to genes supported by many). Entries were mapped to reviewed UniProt [20] entries in order to facilitate analysis in the context of PPI networks. Entries that could not be mapped (e.g. intergenic SNPs) were omitted from the analysis.

#### Amyotrophic Lateral Sclerosis online Database (ALSoD)

ALSoD [21] is a repository combining information genotype, phenotype and geographical information.

Data was downloaded from ALSoD on May 27^th^ 2014. The dataset consisted of 114 genes, out of which 111 could be mapped to 111 reviewed UniProt proteins.

#### ALSGene. ALSGene is a database of genes associated with ALS through GWAS

The ALSGene “Top Results” dataset [22] was downloaded on May 27^th^ 2014. The dataset consists of 22 genetic polymorphisms, out of which 17 were mapped to genes. All 17 genes could be mapped to reviewed UniProt proteins.

#### Genotator

Genotator [23] combines data from 11 external sources to provide information on human diseases.

All data associated with “Amyotrophic Lateral Sclerosis” was downloaded from Genotator on April 30^th^ 2014. The dataset consists of 294 loci, out of which 289 genes could be mapped to 289 reviewed UniProt proteins.

#### Genetic Association Database (GAD)

GAD contains genetic associations from complex diseases.

The entire dataset from GAD [24, 25] was downloaded on May 2^nd^ 2014. Afterwards all genes associated with “Amyotrophic Lateral Sclerosis” (and subcategories) were extracted yielding a dataset consisting of 275 loci, out of which 226 could be mapped to 228 reviewed UniProt proteins.

#### HuGE Navigator, GWAS

The full gene level GWAS [26] dataset was downloaded from the HuGE Navigator website on May 1^st^ 2014. Each line containing the text string “Amyotrophic lateral sclerosis” was extracted yielding a dataset of 76 records which after redundancy reduction and removal of intergenic regions gave rise to a list of 46 genes, out of which 43 could be mapped to reviewed UniProt proteins.

Furthermore, a list of genes in the 200kb regions surrounding ALS-associated SNPs was downloaded on May 6^th^ 2014. Following redundancy reduction 101 genes were indicated, out of which 97 could be mapped to reviewed UniProt proteins.

The 200kb region list was combined with the gene level list to yield a final list of 118 proteins.

#### HuGE Navigator, Gene prospector

A dataset of all genes associated to “Amyotrophic Lateral Sclerosis” was downloaded from the HuGE Navigator website on May 1st 2014 via the “Gene Prospector” search interface [27]. The dataset consisted of 194 genes, out of which 191 could be mapped to reviewed UniProt proteins. In total 224 UniProt proteins were indicated due to mapping of HLA-B to multiple allelic variants.

#### Text mining

Finally, we used the InBio Know [28] text-mining solution from Intomics (see the text mining section below for details) to build a list of genes significantly associated with ALS based on the May 2014 set of PubMed abstracts. The list consisted of 164 proteins (already based on UniProt IDs thus no further mapping was needed).

#### Combined ALS-associated list

All lists of ALS gene/protein associations were aggregated to yield a total list of 656 proteins–see S1 Table.

#### ALS-associated genes added since 2014 data survey

An updated dataset of ALS associated genes were downloaded from ALSoD and HuGE Navigator Genopedia on April 27, 2020. Genes were mapped to UniProt IDs. A new round of text mining was conducted to identify new proteins associated with ALS in the scientific literature.

A total of 10 and 91 new proteins (i.e. not present in the ALS-databases at the time of the original data survey) were found in ALSoD and HuGE Navigator Genopedia, respectively, while 61 new proteins were identified through text mining. Due to overlap between gene sets from the three resources, a total set of 140 unique proteins were found to be associated with ALS since May 2014 (S2 Table).

Proteins present in ALS core modules were tested for overrepresentation of new ALS-associated proteins using a Fisher’s exact test.

For the remaining databases no new datasets were obtained. The ALSGene database has not been updated since 2011 and Genotator and GAD databases have been discontinued.

### Generating ALS-associated PPI networks

The PPI resource inBio Map/InWeb_IM [13] was chosen as the source of PPIs for building a comprehensive collection of ALS related networks. Briefly, inBio Map is a large, robust, high confidence database of inferred human physical PPIs gathered from multiple databases of experimental evidence. Human PPIs were obtained from the February 2014 version of inBio Map. Low confidence interactions were filtered out using a confidence score cutoff of 0.1, resulting in 130,746 high confidence interactions between 11,900 proteins.

The ALS network collection was built by considering all first order networks around the ALS-associated proteins (the aggregated inclusive list, across all datasets, we consider these the “seed proteins”), then pruning the networks for over-connected (proteins with a high number of interactions outside the current network), and finally merging all networks with a high degree of overlap. This yielded a collection of 282 networks with the size distribution shown in S1 Fig. This collection of networks is also available as a Cytoscape [29] data file as part of the supplementary materials.

### Defining ALS core modules

From the full set of 282 networks, we define the sub-set of networks significantly enriched in ALS-associated genes/proteins as the set of **ALS core modules**. Each of the modules in the core set, is more likely to be close to known ALS biology, and has been investigated further with regards to both molecular biology as well as overlap with genes/proteins associated with other diseases, that significantly share these core modules with ALS.

The set of five modules significant after Bonferroni correction are in the following termed ’Tier 1’, while the remaining modules significant only after the less strict Benjamini-Hochberg correction are termed ’Tier 2’. Tier 1 and Tier 2 are collectively referred to as the ‘ALS core modules’.

### Biological annotation of the ALS network collection

We investigated the biology represented in each of the ALS-associated disease modules using two lines of evidence: 1) overrepresentation of Gene Ontology [30, 31] ‘Biological processes’ terms and 2) overlap with other diseases.

In both cases of overrepresentation analysis, the gene/protein identifiers were extracted from the individual ALS-associated modules and used as the study set in the analysis and the entire human interactome as the background set. Fisher’s Exact test was used to test for overrepresentation of GO terms/diseases among the network proteins. The significance threshold was adjusted for multiple testing using Bonferroni correction.

After GO annotation 3 filtering steps were applied to select the most relevant GO terms representing each disease module:

1. GO term must be ‘minimal’. When a p-value for a GO term has been calculated, it is compared to the p-values for all child GO terms. The p-value is then said to be "minimal" if it is less than all the p-values for all the child GO terms (or if the GO term does not have any children).
2. GO term overrepresentation must be significant after Bonferroni correction (p_corr_ < 0.05)
3. GO term must contain < 1000 genes

GO terms were manually curated by biological experts, to remove very generic GO terms (e.g. GO:0022402, “cell cycle process” or GO:0000077 “DNA damage checkpoint”), or irrelevant GO terms based on a biological assessment, resulting in a total of 182 GO terms being overrepresented in the top 26 modules. To reduce the redundancy inherently present in the GO hierarchy (some GO terms have a high degree of overlap, or multiple GO terms can describe the same biology), the 182 GO terms were then manually collected into GO biological classes based on their labels. GO classes were furthermore collected into GO super-classes representing broad biological classes or functions (S3 Table).

### Visualization of networks

All visualizations of disease modules, including the visualization of protein-level metadata (known ALS-associated genes/proteins, biological categories) were performed using Cytoscape [29].

### Text mining of diseases co-mentioned with ALS

The inBio Know text mining software suite [28] was used to find associations between 1) diseases vs. genes and 2) diseases vs. diseases in PubMed abstracts. Manually curated synonyms for all human genes/proteins as well as synonyms for all Disease Ontology [32, 33] diseases were used.

The text mining software was used as follows:

1. For each item of interest (e.g. a human disease) all matches in PubMed for any of its curated synonyms were recorded. Multiple hits in a single PubMed entry would be counted as one. From this the background frequency in the entire pool of PubMed abstract was calculated.
2. For pairs of items of interest (e.g. a disease and a gene/protein), the number of abstracts co-mentioning the terms was counted, and enrichment over background frequencies (observed / expected) was calculated using Fisher’s Exact test.
3. With-in each sub-study (e.g. human disease vs. all human genes/proteins), the significance threshold was adjusted for multiple testing using Bonferroni correction.

#### Disease vs. disease comparison

An initial round of text mining to find diseases co-mentioned with ALS in PubMed abstracts was conducted, and from this we extracted all diseases with at least one co-mentioning with ALS. The significance of each disease/ALS-association was calculated using a Fisher’s exact test. From the total set of diseases, the top 50 most significantly overrepresented hits with respect to the association with ALS were selected as the pool of diseases to investigate further.

#### Disease vs. genes/proteins comparison

Each of the top 50 diseases identified above, were text-mined for co-mentioning with human genes/proteins in PubMed abstracts (S4 Table). A *p*-value threshold of 10^−10^ was used to call a significant association between a disease and a gene/protein—notice that this is even more restrictive that the standard Bonferroni correction of testing a theoretical maximum of up to 20,500 genes/proteins per disease (*p<2*.*4x10*^*-6*^). The 10^−10^ threshold corresponds to the high-confidence subset of disease/gene associations in the inBio Know software suite.

## Results

### Overlap of ALS-associated genes in different data bases

**Fig 2:**
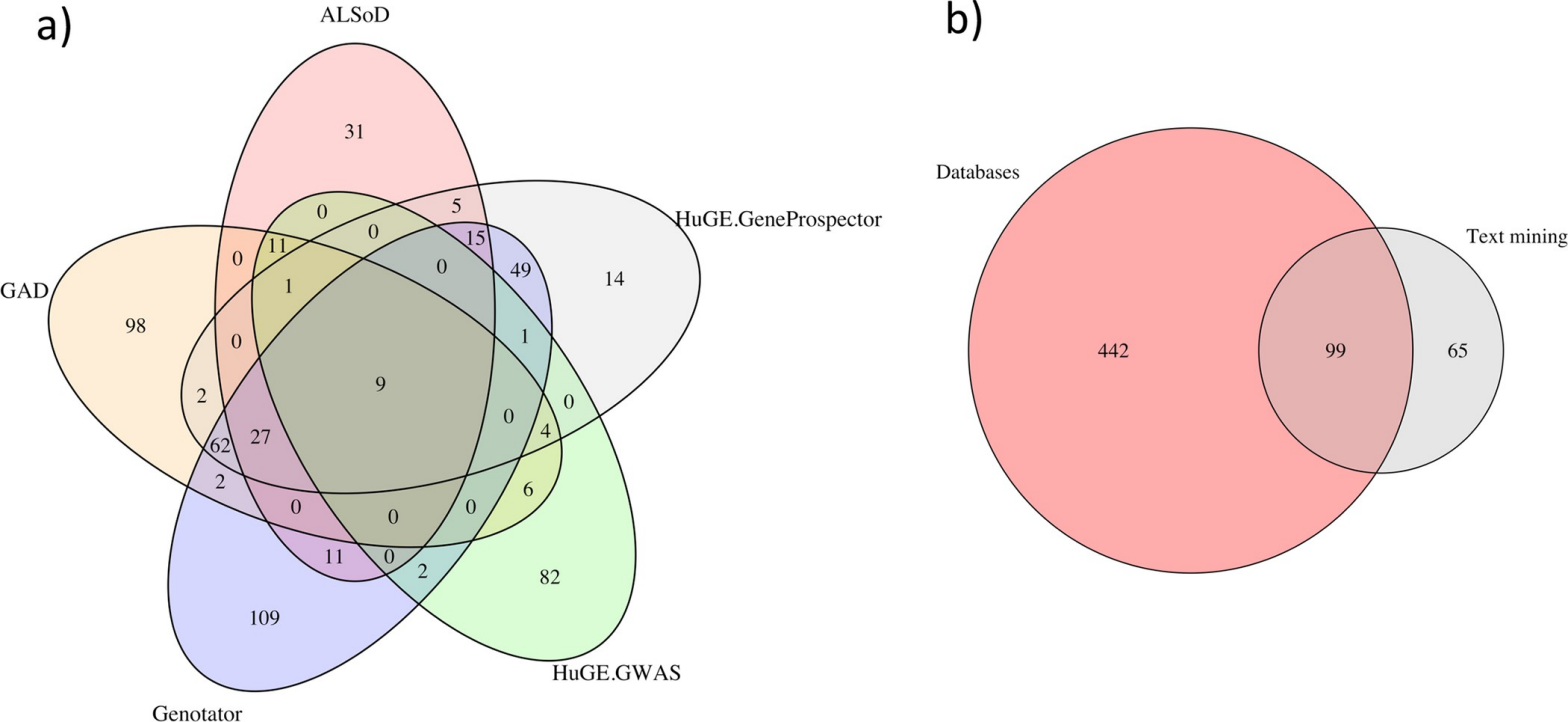
Overlap between ALS resources. Overlap between proteins suggested to be ALS-associated. Panel A: Overlap between the 5 largest ALS databases. Panel B: Overlap between the combined databases and the text mining-based list.

A comprehensive analysis of 7 databases for canonical ALS-associated proteins, yielded 656 proteins linked to ALS (Fig 2, S1 Table). There was a surprisingly low overlap between the ALS-associated proteins obtained from the 7 sources we used to build the dataset (see Fig 2). Even considering the diversity of the sources, this appears to indicate a level of uncertainty whether these genes are truly associated with ALS. A set of only 29 proteins had a high level of agreement in 5 out of 7 data sources (Table 1), indicating the most comprehensively studied subset of ALS related genes. Among these genes are the known players of ALS pathology such as SOD1, C9ORF72, TARDBP, as well as many less well-established genes, which are thought to constitute additional risk factors for causation, modification or progression of ALS (for example SQSTM1 and VCP [4]). Other putative ALS-associated genes are found only in one database or in literature and their contribution to ALS pathogenesis needs to be studied further.

**Table 1:** ALS-associated genes/proteins supported by 5 or more resources.

	Resource							Protein information	
Gene name	ALSGene (n = 17)	ALSoD (n = 111)	GAD (n = 224)	Genotator (n = 289)	HuGE GWAS (n = 118)	HuGE GeneProspector (n = 224)	Text Mining (n = 164)	UniProt entry	Protein name
C9ORF72	•	•	•	•	•	•	•	CI072_HUMAN	Protein C9orf72
DPP6		•	•	•	•	•	•	DPP6_HUMAN	Dipeptidyl aminopeptidase-like protein 6
ITPR2		•	•	•	•	•	•	ITPR2_HUMAN	Inositol 1,4,5-trisphosphate receptor type 2
KIFAP3		•	•	•	•	•	•	KIFA3_HUMAN	Kinesin-associated protein 3
SOD1		•	•	•	•	•	•	SODC_HUMAN	Superoxide dismutase [Cu-Zn]
UNC13A		•	•	•	•	•	•	UN13A_HUMAN	Protein unc-13 homolog A
ALS2		•	•	•		•	•	ALS2_HUMAN	Alsin
ALS6, FUS		•	•	•		•	•	FUS_HUMAN	RNA-binding protein FUS
ANG		•	•	•		•	•	ANGI_HUMAN	Angiogenin
CHMP2B		•	•	•		•	•	CHM2B_HUMAN	Charged multivesicular body protein 2b
CST3		•	•	•		•	•	CYTC_HUMAN	Cystatin-C
DCTN1		•	•	•		•	•	DCTN1_HUMAN	Dynactin subunit 1
DYNC1H1		•	•	•		•	•	DYHC1_HUMAN	Cytoplasmic dynein 1 heavy chain 1
ELP3		•	•	•		•	•	ELP3_HUMAN	Elongator complex protein 3
FGGY		•	•	•		•	•	FGGY_HUMAN	FGGY carbohydrate kinase domain-containing protein
HFE		•	•	•		•	•	HFE_HUMAN	Hereditary hemochromatosis protein
LIPC		•	•	•	•	•		LIPC_HUMAN	Hepatic triacylglycerol lipase
PON1		•	•	•		•	•	PON1_HUMAN	Serum paraoxonase/arylesterase 1
PON2		•	•	•		•	•	PON2_HUMAN	Serum paraoxonase/arylesterase 2
PON3		•	•	•		•	•	PON3_HUMAN	Serum paraoxonase/lactonase 3
PRPH		•	•	•		•	•	PERI_HUMAN	Peripherin
SMN2		•	•	•		•	•	SMN_HUMAN	Survival motor neuron protein
SUSD1		•	•	•	•	•		SUSD1_HUMAN	Sushi domain-containing protein 1
TARDBP		•	•	•		•	•	TADBP_HUMAN	TAR DNA-binding protein 43
VAPB		•	•	•		•	•	VAPB_HUMAN	Vesicle-associated membrane protein-associated protein B/C
VCP		•	•	•		•	•	TERA_HUMAN	Transitional endoplasmic reticulum ATPase
VEGF, VEGFA		•	•	•		•	•	VEGFA_HUMAN	Vascular endothelial growth factor A
VPS54		•	•	•		•	•	VPS54_HUMAN	Vacuolar protein sorting-associated protein 54
ZFP64		•	•	•	•	•		ZF64A_HUMAN	Zinc finger protein 64 homolog, isoforms 1 and 2
ZFP64		•	•	•	•	•		ZF64B_HUMAN	Zinc finger protein 64 homolog, isoforms 3 and 4

29 proteins found in > = 1 data source. 8 out of the most frequent proteins were found in one or more of the 26 ALS core modules.

### ALS-associated genes with network support

Without biological context, individual genes that are identified to be associated with ALS may be useful for diagnosis but do not contribute to the understanding of the molecular pathophysiology and the subsequent search for prevention or treatment [34]. However, if these genes are part of networks which are significantly enriched in ALS-associated proteins, it can help to reinforce the evidence for more weakly supported proteins. To investigate this further, we evaluated a collection of 282 PPI network modules for overrepresentation of ALS-associated proteins (see Methods for details). 26 ALS modules were significantly enriched for ALS-associated proteins after multiple testing correction using the Benjamini-Hochberg procedure (q < 0.1). Five of these were also significant after correcting for multiple testing using Bonferroni correction (p < 0.05/282). The set of five modules significant after Bonferroni correction are in the following termed ’Tier 1’, while the remaining 21 modules are termed ’Tier 2’. Tier 1 and Tier 2 modules are collectively referred to as ‘ALS core modules’.

36 ALS-associated genes/proteins are supported by Tier 1 modules (Table 2). Nine of these proteins were only mentioned in one source, often found only by text mining. The link to Tier 1 ALS core modules strengthens the likelihood that these genes are indeed ALS-relevant genes. Further 108 genes/proteins have Tier 2 support for a total of 144 genes/proteins having Tier 1/2 network support (see S1 Table for full list).

**Table 2:** ALS-associated proteins in Tier 1 modules.

	Resource								Protein information	
Gene name	ALSGene (n = 17)	ALSoD (n = 111)	GAD (n = 224)	Genotator (n = 289)	HuGE GWAS (n = 118)	HuGE GeneProspector (n = 224)	Text Mining (n = 164)	Overlap	UniProt entry	Protein name
SOD1		•	•	•	•	•	•	6	SODC_HUMAN	Superoxide dismutase [Cu-Zn]
DCTN1		•	•	•		•	•	5	DCTN1_HUMAN	Dynactin subunit 1
ALS6, FUS		•	•	•		•	•	5	FUS_HUMAN	RNA-binding protein FUS
LIPC		•	•	•	•	•		5	LIPC_HUMAN	Hepatic triacylglycerol lipase
TARDBP		•	•	•		•	•	5	TADBP_HUMAN	TAR DNA-binding protein 43
VCP		•	•	•		•	•	5	TERA_HUMAN	Transitional endoplasmic reticulum ATPase
APOE		•	•	•		•		4	APOE_HUMAN	Apolipoprotein E
ATXN2		•		•		•	•	4	ATX2_HUMAN	Ataxin-2
SQSTM1		•		•		•	•	4	SQSTM_HUMAN	Sequestosome-1
MAPT		•		•		•	•	4	TAU_HUMAN	Microtubule-associated protein tau
UBQLN2		•		•		•	•	4	UBQL2_HUMAN	Ubiquilin-2
APOA4			•	•		•		3	APOA4_HUMAN	Apolipoprotein A-IV
LPA			•	•		•		3	APOA_HUMAN	Apolipoprotein(a)
APOB			•	•		•		3	APOB_HUMAN	Apolipoprotein B-100
APOC3			•	•		•		3	APOC3_HUMAN	Apolipoprotein C-III
CCS		•		•			•	3	CCS_HUMAN	Copper chaperone for superoxide dismutase
CETP			•	•		•		3	CETP_HUMAN	Cholesteryl ester transfer protein
CNTF		•		•			•	3	CNTF_HUMAN	Ciliary neurotrophic factor
LDLR			•	•		•		3	LDLR_HUMAN	Low-density lipoprotein receptor
LPL			•	•		•		3	LIPL_HUMAN	Lipoprotein lipase
RNF19A		•		•			•	3	RN19A_HUMAN	E3 ubiquitin-protein ligase RNF19A
HNRNPA1		•				•	•	3	ROA1_HUMAN	Heterogeneous nuclear ribonucleoprotein A1
CHGB			•	•		•		3	SCG1_HUMAN	Secretogranin-1
UBQLN1				•		•	•	3	UBQL1_HUMAN	Ubiquilin-1
ARHGEF28, RGNEF		•				•		2	ARG28_HUMAN	Rho guanine nucleotide exchange factor 28
P4HB						•	•	2	PDIA1_HUMAN	Protein disulfide-isomerase
BCL2							•	1	BCL2_HUMAN	Apoptosis regulator Bcl-2
CASP3							•	1	CASP3_HUMAN	Caspase-3
DERL1							•	1	DERL1_HUMAN	Derlin-1
HDAC6							•	1	HDAC6_HUMAN	Histone deacetylase 6
HECW1							•	1	HECW1_HUMAN	E3 ubiquitin-protein ligase HECW1
HS3ST3A1			•					1	HS3SA_HUMAN	Heparan sulfate glucosamine 3-O-sulfotransferase 3A1
MYLIP			•					1	MYLIP_HUMAN	E3 ubiquitin-protein ligase MYLIP
NEFM							•	1	NFM_HUMAN	Neurofilament medium polypeptide
PARK7				•				1	PARK7_HUMAN	Protein deglycase DJ-1
SORT1							•	1	SORT_HUMAN	Sortilin

36 ALS-associated proteins present in at least of the five Tier 1 networks. A full list of ALS-associated proteins found in > = 1 of the ALS core modules is found in S1 Table.

An effort to identify new ALS-associated proteins through a combination of text mining and database searches revealed a set of 140 proteins not present in the initial data survey (S2 Table). 17 (12.1%) new proteins are found in one or more Tier1 + Tier2 disease modules, which is a significant overlap (p = 0.03).

### ALS network collection

The collection of all 282 ALS-associated PPI networks, offers the opportunity to investigate the biology of networks closely associated with ALS related genes, as well as a framework for mapping experimental data (e.g. gene expression data) to the networks. The networks are available for download as a Cytoscape session file as part of the supplementary materials. A separate Cytoscape session is available for download containing only the **ALS core modules**, with Tier 1 vs. Tier 2 clearly marked in the overview. Furthermore, it contains all metadata and graphical styles needed to generate the visualization of the disease modules shown in this publication, thus allowing for further exploration of the **ALS core modules**.

### ALS core modules in overview

**Fig 3:**
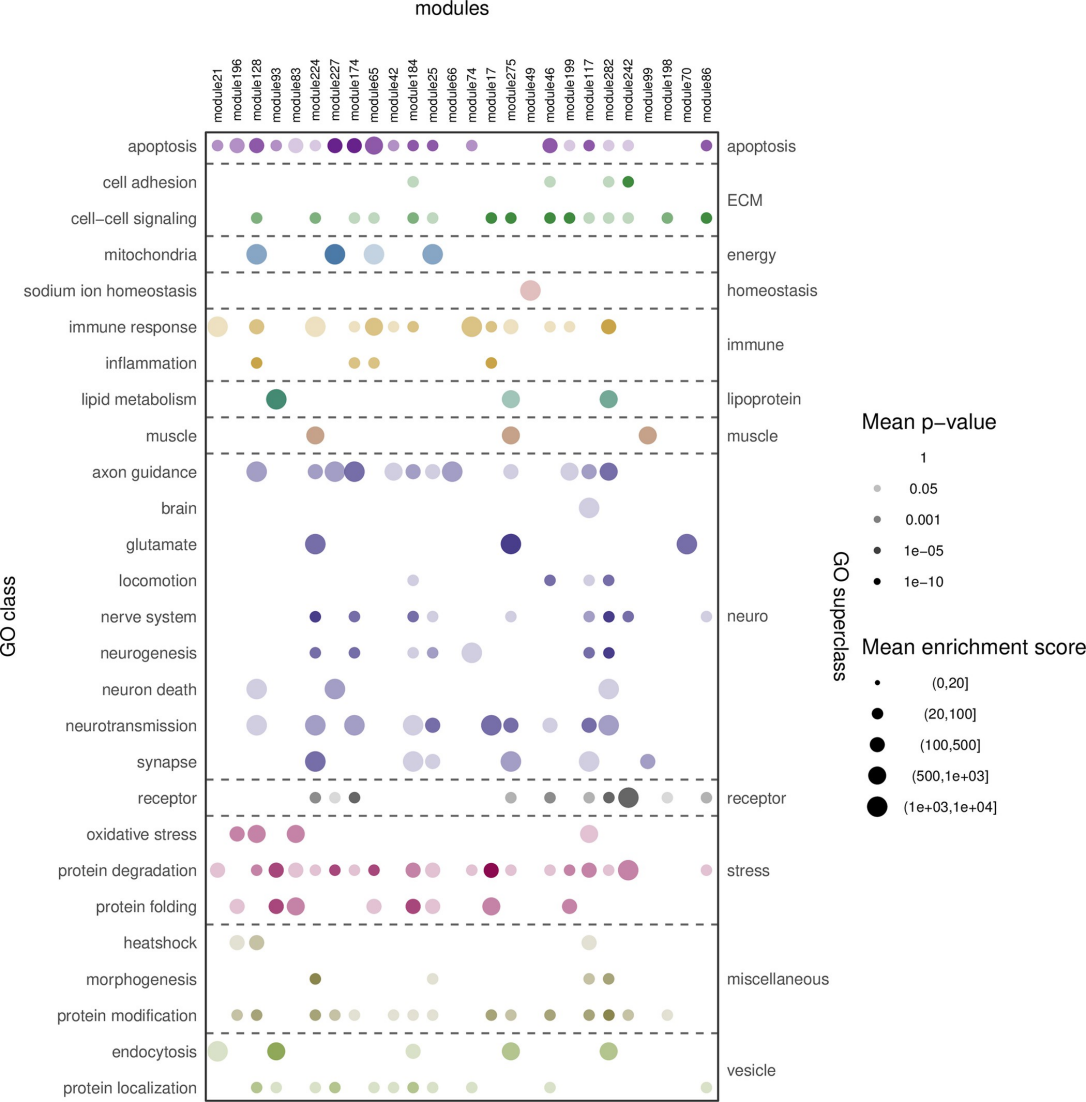
Relevant Gene Ontology categories overrepresented in ALS core modules. Top row: Module IDs–sorted on significance of ALS overrepresentation (most significant to the left). Left: Significantly overrepresented Gene Ontology categories were manually collected in GO classes. GO class significance and enrichment score is calculated as the geometric mean of the p-values and mean of the enrichment scores, respectively, for all contained GO categories. Right: GO super-classes is a top-level descriptor for the GO classes and categories.

Investigating the spectrum of molecular biology represented in the 26 **ALS core modules** (Fig 3), by evaluating the Gene Ontology categories overrepresented in them, leads to the following observations: *Apoptosis is* represented in most (19) of the modules, as is *protein degradation* (19). A large proportion (19) of the modules are enriched for genes/proteins involved in *protein- modification* (15) or *-localization* (11). *Axon guidance*, and *immune response* are represented in 12 and 13 core modules, respectively.

Some GO terms were only represented in Tier 2 core modules and not in Tier 1 modules. These include GO terms are centered around muscle, nervous system, synapse and glutamate, which are classically linked to ALS.

With a focus on the five Tier 1 core modules this analysis showed that most core modules are representing apoptosis and most often linking it to protein degradation or core module specific additional GO terms, for example core module 93 exclusively contains *lipoprotein*.

**Fig 4:**
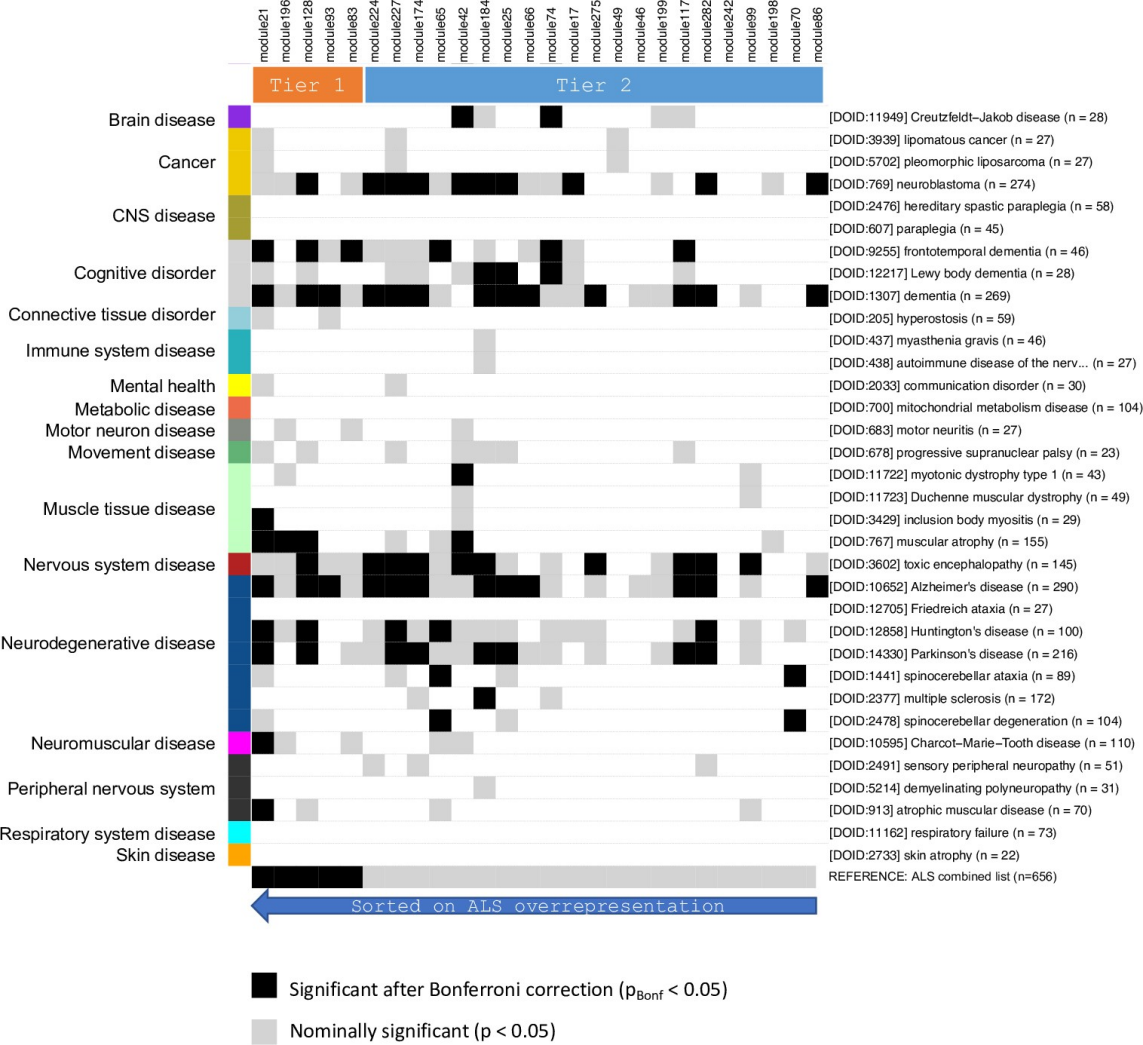
Disease overlap matrix for ALS core modules. Top row: Module IDs–sorted on significance of ALS overrepresentation (most significant to the left), and Tier 1 / Tier 2 subsets indicated. Right side: Disease description: Disease Ontology ID, name (truncated to 35 letters), number of genes/proteins associated to the disease via text mining. Only diseases with a least 20 associated genes/proteins included in this figure. Left: Disease categories as defined in Table 3.

Based on the 50 diseases most significantly co-mentioned with ALS in PubMed abstracts (Table 3) an overrepresentation analysis was performed. Disease-associated genes were then overlapped with the ALS core modules to identify connections to other diseases. A total of 37 diseases were overlapping with at least 1 ALS core module (Fig 4).

**Table 3:** Top 50 diseases most commonly co-mentioned with ALS in PubMed abstracts. List of the 50 diseases most commonly co-mentioned with ALS in scientific literature. Diseases have manually been categorized into ‘Disease types’ guided by the tree structure in Disease Ontology [32, 33]. ID: Disease Ontology ID. Abbreviation: Short name for disease used in Figs 5–9. Name: Disease name in Disease Ontology. Occurences: Number PubMed abstracts mentioning disease. Co-mentionings: Number of PubMed abstracts where both disease and ALS are mentioned. Overrep. ratio: Overrepresentation ratio of co-mentionings. P-value: Significance of hypergeometric test of the overlap between disease and ALS. Disease type: Manual classification of diseases.

ID	Abbreviation	Name	Occurrences	Co-mentionings	Overrep. ratio	P-value	Disease type
DOID:11949	CJD	Creutzfeldt-Jakob disease	4,508	61	14.6	1.59E-48	Brain disease
DOID:12680	PseudoBulbPalsy	pseudobulbar palsy	392	22	60.5	7.41E-32	
DOID:12859	Chorea	choreatic disease	3,001	38	13.7	7.75E-30	
DOID:5702	PleoLiposac	pleomorphic liposarcoma	4,293	69	17.3	1.54E-59	Cancer
DOID:3939	LipoCanc	lipomatous cancer	4,378	69	17.0	5.59E-59	
DOID:769	NeuroBlast	neuroblastoma	27,962	99	3.8	5.56E-28	
DOID:2476	HS Paraplegia	hereditary spastic paraplegia	1,690	103	65.7	8.73E-147	Central nervous system disease
DOID:607	Paraplegia	paraplegia	9,017	114	13.6	5.10E-86	
DOID:1307	Dementia	dementia	136,817	2,639	20.8	~0	Cognitive disorder
DOID:9255	FT Dementia	frontotemporal dementia	5,737	1,217	228.8	~0	
DOID:12217	LB Dementia	Lewy body dementia	3,916	81	22.3	4.24E-78	
DOID:5408	PBD	Paget’s disease of bone	1,532	54	38.0	6.84E-65	Connective tissue disease
DOID:205	BoneHyp	hyperostosis	6,705	54	8.7	5.47E-32	
DOID:4953	Poliomyelitis	poliomyelitis	7,184	95	14.3	1.04E-73	Infectious disease
DOID:4952	PPMSyn	postpoliomyelitis syndrome	535	35	70.6	4.20E-52	
DOID:438	AIDNeuro	autoimmune disease of the nervous system	14,913	189	13.7	1.10E-141	Immune system disease
DOID:12842	GBSyn	Guillain-Barre syndrome	6,675	106	17.1	5.36E-90	
DOID:437	MyaGrav	myasthenia gravis	8,408	105	13.5	7.67E-79	
DOID:2033	ComDis	communication disorder	26,572	129	5.2	9.33E-50	Mental health
DOID:0060046	Aphasia	aphasia	9,060	57	6.8	2.56E-28	
DOID:700	MitoMetaD	mitochondrial metabolism disease	5,245	45	9.3	4.82E-28	Metabolic disease
DOID:683	MotorNeu	motor neuritis	1,915	139	78.3	2.97E-208	Motor neuron disease
DOID:681	ProgBulbPalsy	progressive bulbar palsy	432	97	242.2	1.37E-196	
DOID:0060161	SBMA	Kennedy’s disease	787	96	131.6	6.91E-167	
DOID:678	ProgSupraPalsy	progressive supranuclear palsy	2,782	119	46.1	7.26E-151	Movement disease
DOID:767	MuscAtrophy	muscular atrophy	14,482	897	66.8	~0	Muscle tissue disease
DOID:3429	IncBodyMyositis	inclusion body myositis	1,525	88	62.2	1.52E-123	
DOID:11722	MyoDystT1	myotonic dystrophy type 1	3,772	74	21.2	7.35E-70	
DOID:11723	DuchMuscDys	Duchenne muscular dystrophy	7,844	82	11.3	4.30E-56	
DOID:12858	HD	Huntington’s disease	11,696	642	59.2	~0	Neurodegenerative disease
DOID:14330	PD	Parkinson’s disease	69,957	1,725	26.6	~0	
DOID:10652	AD	Alzheimer’s disease	92,611	1,658	19.3	~0	
DOID:2377	MS	multiple sclerosis	49,375	686	15.0	~0	
DOID:2478	SC Degen	spinocerebellar degeneration	8,342	232	30.0	1.35E-249	
DOID:1441	SC Ataxia	spinocerebellar ataxia	7,104	211	32.0	4.70E-233	
DOID:11870	PickD	Pick’s disease	976	65	71.8	7.94E-96	
DOID:9277	PC Degen	primary cerebellar degeneration	1,134	48	45.7	1.14E-61	
DOID:12705	FR Ataxia	Friedreich ataxia	2,119	56	28.5	2.50E-60	
DOID:10595	CMTD	Charcot-Marie-Tooth disease	8,334	647	83.7	0	Neuromuscular disease
DOID:3602	ToxEnceph	toxic encephalopathy	26,094	293	12.1	1.44E-204	Nervous system disease
DOID:12697	LockedInSyn	locked-in syndrome	5,900	72	13.2	6.12E-54	
DOID:913	AtroMuscD	atrophic muscular disease	5,802	575	106.9	~0	Peripheral nervous system
DOID:5214	DemyelinPN	demyelinating polyneuropathy	3,391	69	22.0	2.59E-66	
DOID:5213	CIDMPRN	chronic inflammatory demyelinating polyradiculoneuropathy	2,061	51	26.7	1.14E-53	
DOID:4308	PR Neuropathy	polyradiculoneuropathy	9,689	74	8.2	1.09E-41	
DOID:2491	SP Neuropathy	sensory peripheral neuropathy	3,520	41	12.6	1.12E-30	
DOID:11162	Resp. Failure	respiratory failure	57,385	418	7.9	1.53E-220	Respiratory system disease
DOID:2733	Skin Atrophy	skin atrophy	12,885	80	6.7	1.16E-38	Skin disease
DOID:318	ProgMusc atrophy	progressive muscular atrophy	303	141	501.9	~0	Spinal cord disease
DOID:0050881	IBMPFD	inclusion body myopathy with Paget disease of bone and frontotemporal dementia	105	26	267.1	3.84E-55	Syndrome

From the matrix of disease overrepresentation in ALS core modules some clear trends are seen. First of all, the well-known ALS comorbidity *Dementia* is strongly evident from the matrix: *Dementia*, *broad term* (13 modules), the clinically closely associated *Frontotemporal Dementia* (6 modules, 3 of which Tier 1) and *Lewy Body Dementia* (3 modules). Among the group of other nervous system diseases, the following conditions are also associated with the ALS core modules: *Alzheimer’s Disease* (12 modules of which 3 are in Tier 1), *Parkinson’s Disease* (8 modules), *Huntington’s Disease* (5 modules) and *Muscular Atrophy* (4 modules, 3 of which are in the Tier 1 collection). The remaining diseases have 2 or fewer modules associated–including *Multiple Sclerosis* with only 1 module (184), being significantly associated. The only other non-degenerative CNS-diseases being prominently represented by mostly overlapping ALS core modules are *neuroblastoma* (10 modules) and *toxic encephalopathy*, which is likely due to the many modules described by *apoptosis* GO terms and containing a significant enrichment of brain-associated proteins. It is interesting to note, that motor diseases (such as spastic paraplegia, paraplegia, and Friedreich’s ataxia) are not represented by any of the ALS core modules, while muscle diseases, such as atrophic muscular disease, muscular atrophy, myotonic dystrophy type 1 and inclusion body myositis are significantly represented by at least one ALS core module.

### ALS-Tier 1 core modules

**Fig 5:**
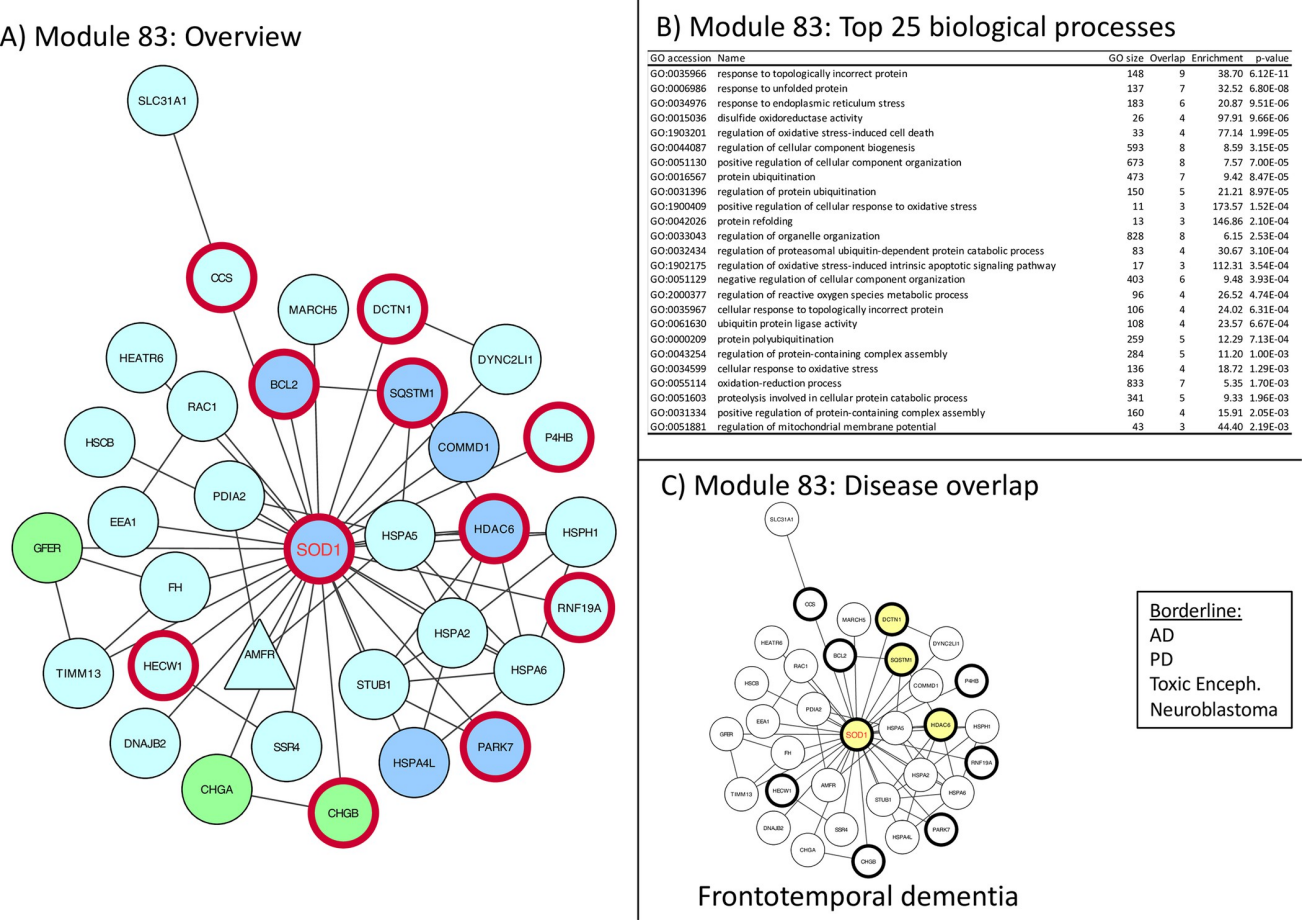
Module 83 –Protein folding and oxidative stress. Panel A: thick red border: gene/protein annotated to be ALS-associated on any of the 7 lists. Large red label: gene/protein with GWAS evidence. Triangular shaped nodes: receptors (UniProt keyword). Node colors indicate subcellular location (UniProt keywords): Blue = Nuclear, Green = Secreted, Purple = conflicting nuclear/secreted, cyan = neither nuclear nor secreted (likely cytosolic). Panel B: Gene Ontology overrepresentation analysis. Panel C: Thick border: ALS-associated (as panel A), Colored node: Disease-associated gene/protein.

**Fig 6:**
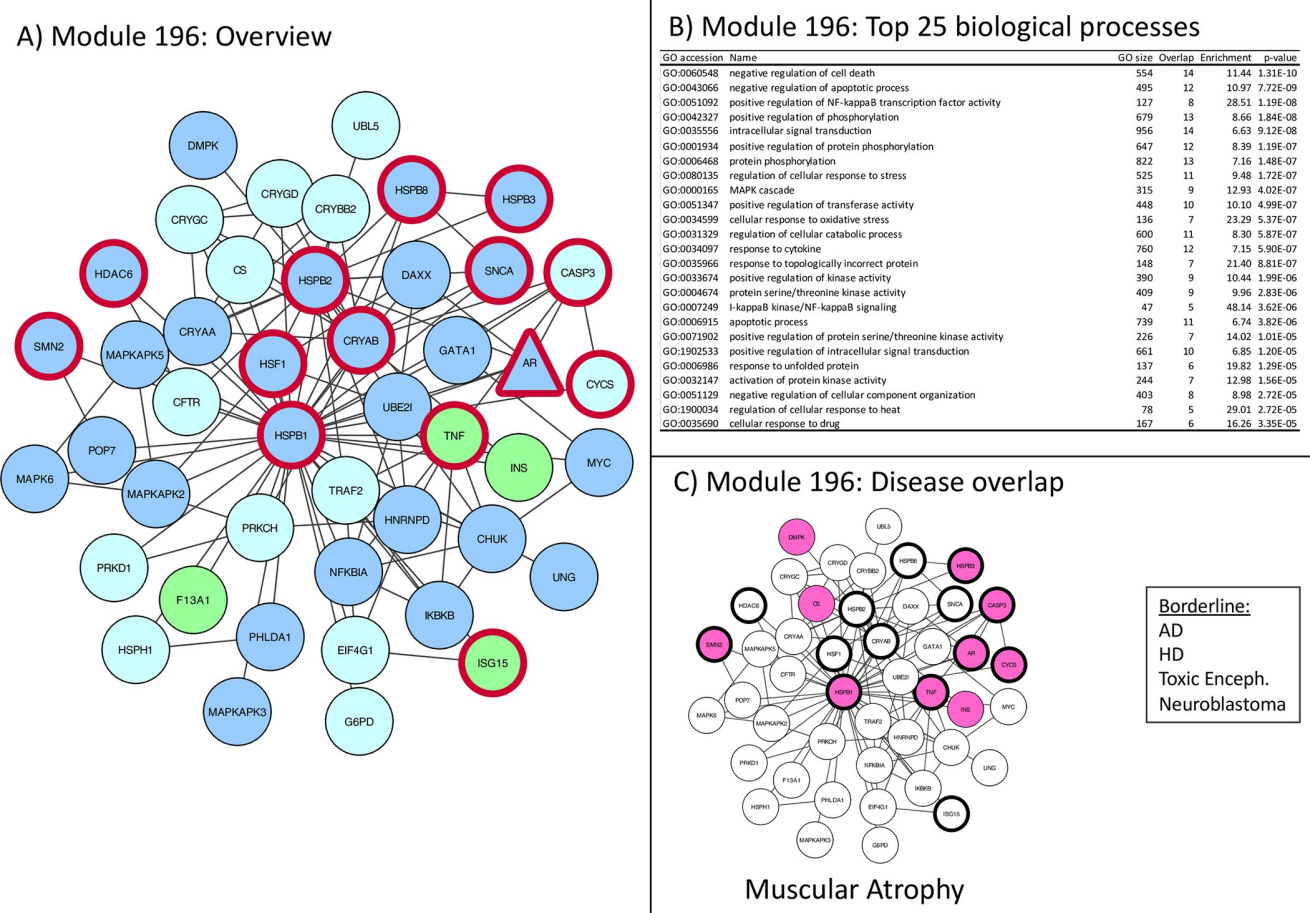
Module 196 –apoptosis and oxidative stress related. Panel A: thick red border: gene/protein annotated to be ALS-associated on any of the 7 lists. Large red label: gene/protein with GWAS evidence. Triangular shaped nodes: receptors (UniProt keyword). Node colors indicate subcellular location (UniProt keywords): Blue = Nuclear, Green = Secreted, Purple = conflicting nuclear/secreted, cyan = neither nuclear nor secreted (likely cytosolic). Panel B: Gene Ontology overrepresentation analysis. Panel C: Thick border: ALS-associated (as panel A), Colored node: Disease-associated gene/protein.

**Fig 7:**
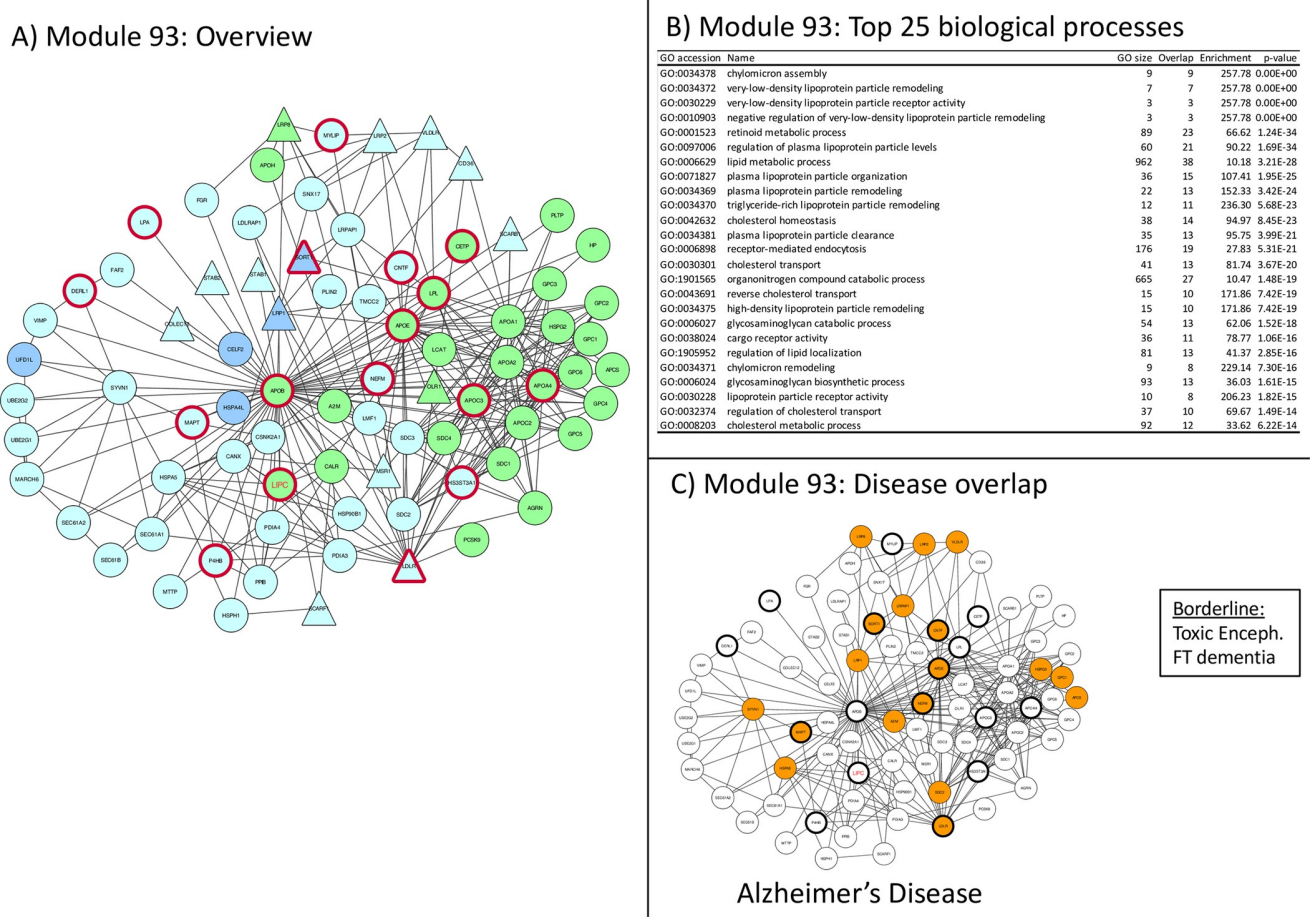
Module 93 –lipid metabolism related. Panel A: thick red border: gene/protein annotated to be ALS-associated on any of the 7 lists. Large red label: gene/protein with GWAS evidence. Triangular shaped nodes: receptors (UniProt keyword). Node colors indicate subcellular location (UniProt keywords): Blue = Nuclear, Green = Secreted, Purple = conflicting nuclear/secreted, cyan = neither nuclear nor secreted (likely cytosolic). Panel B: Gene Ontology overrepresentation analysis. Panel C: Thick border: ALS-associated (as panel A), Colored node: Disease-associated gene/protein.

**Fig 8:**
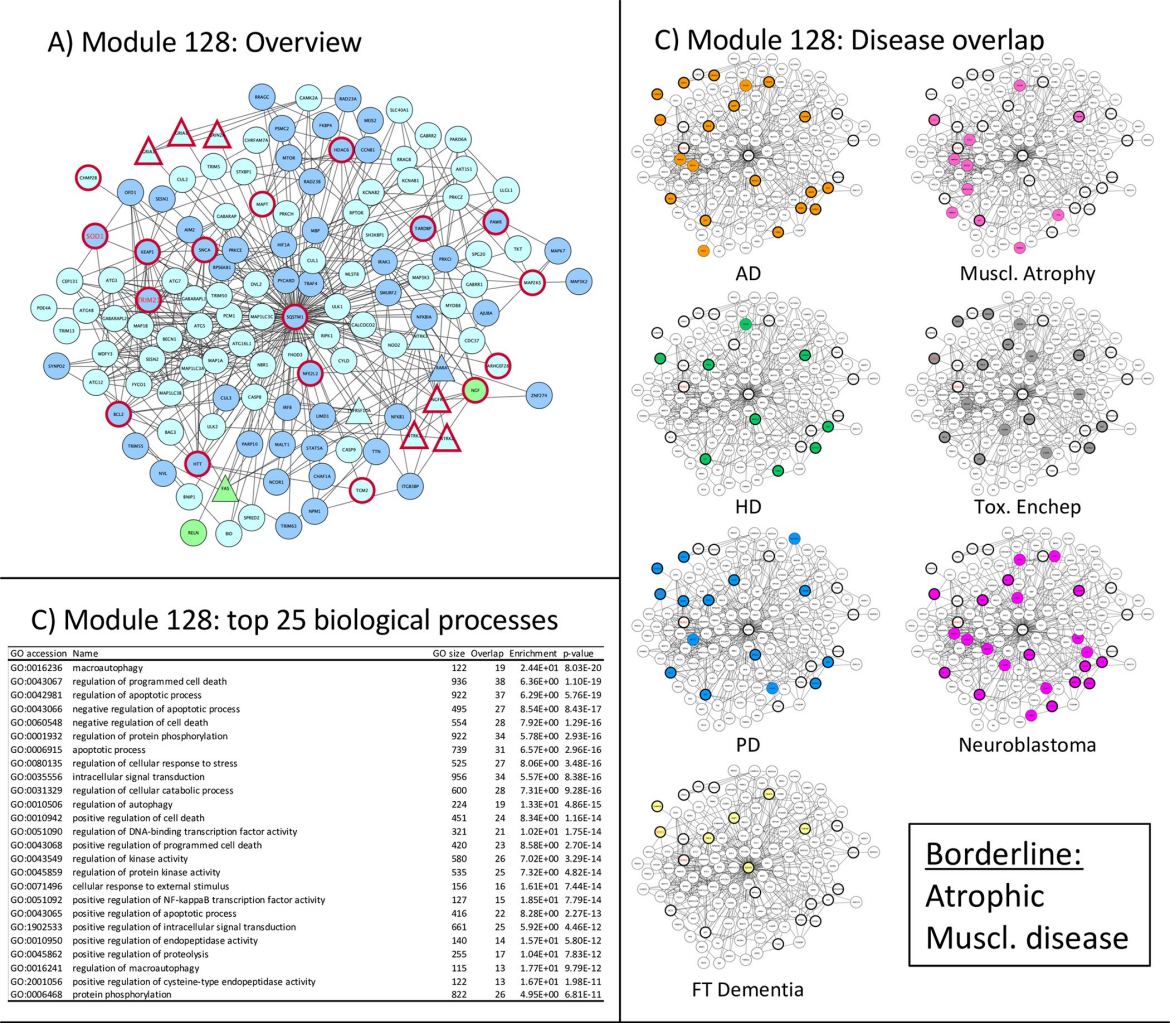
Module 128 –stress and apoptosis related. Panel A: thick red border: gene/protein annotated to be ALS-associated on any of the 7 lists. Large red label: gene/protein with GWAS evidence. Triangular shaped nodes: receptors (UniProt keyword). Node colors indicate subcellular location (UniProt keywords): Blue = Nuclear, Green = Secreted, Purple = conflicting nuclear/secreted, cyan = neither nuclear nor secreted (likely cytosolic). Panel B: Gene Ontology overrepresentation analysis. Panel C: Thick border: ALS-associated (as panel A), Colored node: Disease-associated gene/protein.

**Fig 9:**
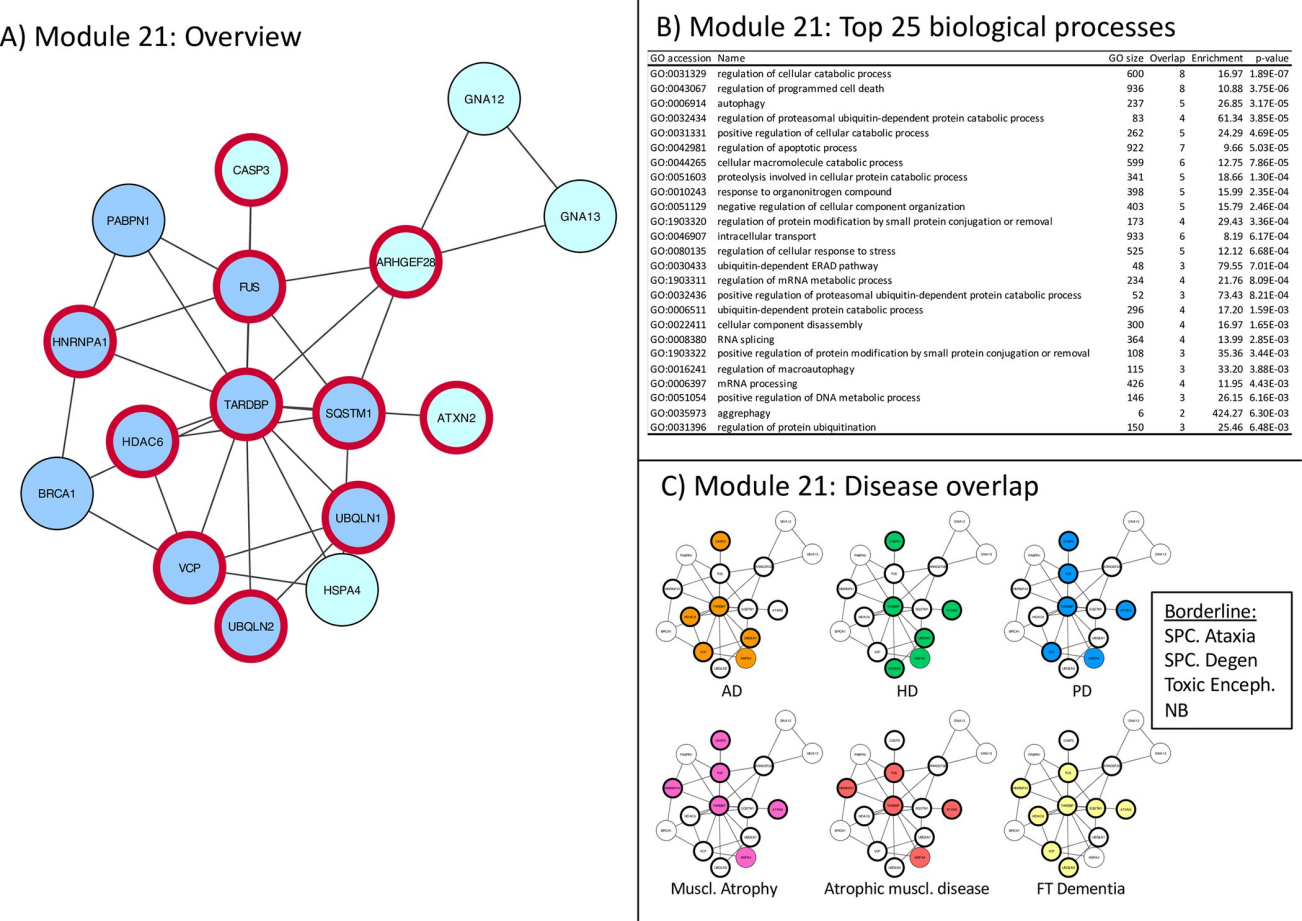
Module 21 –weakly classified biology, strong disease overlap. Panel A: thick red border: gene/protein annotated to be ALS-associated on any of the 7 lists. Large red label: gene/protein with GWAS evidence. Triangular shaped nodes: receptors (UniProt keyword). Node colors indicate subcellular location (UniProt keywords): Blue = Nuclear, Green = Secreted, Purple = conflicting nuclear/secreted, cyan = neither nuclear nor secreted (likely cytosolic). Panel B: Gene Ontology overrepresentation analysis. Panel C: Thick border: ALS-associated (as panel A), Colored node: Disease-associated gene/protein.

The Tier 1 core modules were then investigated for known ALS disease biology and proteins associated with other neurodegenerative diseases (Figs **5–9**).

**Module 83** –very ALS specific, contains SOD1, links oxidative stress and protein folding, (Fig 5).

The identification of causative mutations in SOD1 gene was the first evidence of genetically inherited forms of ALS [35]. SOD1, with its many mutations is therefore the best studied protein in this disease and has been linked to two main pathogenic mechanisms which are thought to lead to ALS pathology. Both potential mechanisms are reflected in the underlying biology represented in this network. Mutations in SOD1, a ubiquitously expressed peroxide dismutase, have been linked to oxidative stress, either by a gain of function of this catabolic enzyme or also as a direct regulator of the NADPH dependent oxidation of RAC1 [36]. The network contains many other proteins playing part in the oxidative stress response, therefore the main GO term associated with this network is *oxidative stress* (Fig 5B). Alternatively, mutations in SOD1 have been reported to induce its misfolding and aggregation (GFER, CCS, PDIA2) and thus to lead to loss of function [37]. Protein misfolding elicit a number of cellular mechanisms to protect the cell against the accumulations of aggregates. Representative of these rescue mechanisms are the large number of heat shock chaperones (for example HSPH1, HSPA2-6, DNAJB2) [38], where PARK7 is by itself redox sensitive. Ubiquitin ligases are also present in the proteasomal pathways (HECW1, STUB1, RNF19a). In the **Tier 1 collection**, module 83 is the most specific to ALS and shows minimal overlap with other neurological diseases. Changes in SOD1 associated function leading to a concomitant deficit in proteostasis may therefore be a unique feature of ALS pathology and its close relative frontotemporal dementia.

**Module 196** Represented also in muscular atrophy, linked to protein degradation and apoptosis (F)

Represented also in muscular atrophy, linked to protein degradation and apoptosis (Fig 6)

Module 196 is centered around HSPB1 (HSP27), which has a variety of functions relevant to ALS. This network shows a molecular link to HSPB1 to the crystallin chaperones, which are ZN^2+^ dependently activated and upregulated in neurological diseases [39]. Crystallin chaperones are also associated with myopathies consistent with their abundant expression in muscle where they stabilize Desmins [40]. HSPB1 oligomerization induced by stress, also TNF induced inflammatory stress. The TNF induced apoptotic signaling pathway is activated through MAPKAP, where HSPB1 deactivates DAXX [41]. Apart from its role in apoptosis, HSPB1 is also important in the proper function of proteasomes and can modulate reactive oxygen species. With this focus on responses to oxidative (and inflammatory stress) this network remains specific for ALS and with its interactive link to the crystallin chaperones makes the muscle particularly sensitive to dysregulation. This is reflected in the link of this network to muscular atrophy and Charcot Marie Tooth disease (Fig 6) another neuropathy which is characterized by progressive muscular loss and genetic link to HSPB1 [42].

**Module 93** Represented also in Alzheimer’s disease, *linked to lipoproteins and lipid metabolism* (Fig 7)

Module 93 is the only Tier 1 network significantly linked to lipid metabolism (Fig 7, panel B) through the presence lipoprotein receptors (LPRx), which are part of the cholesterol pathway genes as well as the APO protein family. Lipids and Lipoproteins are implicated in a whole range of biological process, where they are involved as energy substrates, building blocks, structural machinery and bioactive molecules [43]. In ALS, and AD, lipid metabolism has been thought to underlay denervation, mitochondrial dysfunction, excitotoxicity neural transport, cytoskeletal defect and impaired neurotransmitter release [43]. In the context of ALS, the energy metabolism, in particular, may have increased needs and in muscle a switch from glucose to lipid energy has been described [44], as well as changes in glycosphingolipids [45]. In addition, the brain strongly depends on fatty acid oxidation [46]. High fat and ketogenic diet in animals prolonged survival, while caloric restriction was detrimental in SOD1 transgenic mice [47, 48]. Therapeutically, this hypothesis has been tested with a high fat diet in a small clinical trial, which suggests that nutritional intervention needs to be followed up [49].

The high number of APO and LRP proteins in this network potentially drives the significant association with Alzheimer’s Disease, for which genotype of APOE is the main risk factor. While APOE in Alzheimer has been proposed to play a role in many processes [50], we suggest, based on this ALS core module, that its role in CNS lipid homeostasis is similar in ALS and AD, The use of high fat diets in AD has been discussed controversially, however.

**Module 128** –Represented in many neurodegenerative diseases linked to protein metabolism and apoptosis (Fig 8).

Module 128 represents a large network that contains a wide range of proteins. It is overlapping with 83 (SOD1, HDAC6, BCL2, SQSTM1)) as well as with 196 (SNCA, MAPT) as well as with 21 (HDAC6, SQSTM1, TARDP). The high degree of disease overrepresentation in this network may be due to the fact that it is the only network that has neuronal function associated proteins, such as the GABA receptors (GABAx), Glutamate receptors (GRIA1,3, GRIN2a) and neuronal related growth factors (NGF) and the microtubular system (HTT, MAPs). Interestingly, however, it is not associated with multiple sclerosis, suggesting that the dysfunction seen in the clinical presentation of MS is more strongly driven by a different mechanism such as immune dysfunction.

Similar to the other networks, this network contains proteins involved in ubiquitination (KEAP, TRIMs). As diseases are often caused by disturbance of homeostatic functions, these stress networks are found in many diseases activated, which may make this network so important also in non-degenerative diseases, such as neuroblastoma.

**Module 21** –Represented in many neurodegenerative diseases, highest density of ALS genes, but little significant biology (Fig 9).

This network is almost exclusively made up of ALS-associated genes. It directly links many ALS-risk genes (HDAC6, VCP, HNRNPA1, SQSTM1, ATXN2) into the same network with the major causative genetic mutations (TARDBP and FUS). In fact, mutations in most of the proteins in this network have been proposed to be linked in one or the other way to ALS. This may strengthen the importance of these genes in the overall ALS pathogenesis [51]. This module *functionally* links many ALS-associated genes into one network which may partly explain why such a large variety of mutations and risk factors lead to the same pathological and clinical features. This network links the two major pathogenetic theories about ALS that are currently discussed: defects in RNA processing [52] and proteasomal malfunction. Dysfunctional mRNA processing, in addition to loss of function of these transcripts, may lead to an overload of the protein degradation system and thus to cellular dysfunction, independent of the aggregate per se. The finding that TDP-43 (the protein product of TARDBP), is also involved in low molecular weight neurofilament processing and aggregation [53], represents a very interesting insight into how general biological principles can become organ-, here neuron, specific pathologies. In recent years, many neurodegenerative diseases have been recognized and grouped as proteinopathies. Apart from the RNA-related malfunction that comes with mutations of FUS and TARDBP, some studies have recently suggested that these proteins contain prion like structures [54], which makes them prone to seeding and aggregation with other proteins or lead to dysfunction of the protein degradation pathway causing other proteins to aggregate [55]. In particular TDP43 is also found aggregated in other neurodegenerative diseases [56]. This module is therefore strongly associated with other neurodegenerative diseases that have protein deposits (Fig 9C).

## Discussion

While there is a high rate of new genes that become described as potentially relevant to ALS-disease pathogenesis, their role often remains unclear and confirmation is lacking. Network analysis is one way to link individual proteins into functional networks. Being part of a functional network with a biological relevance for ALS may strengthen the association of a described ALS gene to the disease. In this study we identified 656 proteins that have previously been associated with ALS. Of these, 144 genes were connected in 26 ALS core modules, suggesting a functional association with ALS. Some of the previously less well described genes, such as HDAC6 and SQSTM were shown to be part of the majority of the Tier 1 modules, linking them closely to the well-known ALS genes TARDBP, FUS and SOD1.

Several studies have utilized PPI networks in order to understand the biological complexity underlying ALS. In a 2017 study, Mao et al. [17] found proteins connected to known ALS-causative genes in order to identify common downstream proteins. However, despite the apparent overlap with our approach there are some key differences allowing us to reduce the number of false-positive hits and home in on a much more specific biological interpretation.

Using a bottom-up approach, where networks are generated by including first-order neighbors of the ALS associated proteins and merging highly overlapping networks, provides us with a set of distinct disease modules with a well-defined biological annotation.

Another important difference is the consideration of local degree/global degree ratio in order to handle the problem of over-connected proteins. Highly connected proteins (e.g. UBC) will have a higher chance of showing up in any given network than less connected proteins and are known to introduce noise in network analyses. Filtering out noisy proteins gives a much clearer picture of the key proteins involved in any particular biological process (see Introduction for further details).

A wide range of cellular processes have been implicated in ALS pathogenesis, as reviewed recently [57]. These include neuronal-specific processes, including hyper- and hypo-excitability, glutamate excitotoxicity, and neuronal branching defects [58], proteostasis pathways with impairments in ubiquitin–proteasome systems, autophagy and lysosomal function as well as dysfunction in the endoplasmic reticulum (ER) and mitochondria [59]. More recently, altered RNA processing/metabolism, RNA splicing transcriptional defects have been shown to be linked in particular to TARDBP [60]. Furthermore, dysregulation of cytoskeletal dynamics, leading to impairment of vesicular trafficking including nuclear-cytoplasmic transport [61], between ER and Golgi [62], as well as transport along axons [63] have been found to be part of the ALS pathology.

The highly significant networks, represented in the Tier 1 and Tier 2 modules which we presented in more detail in this study, capture many of these pathophysiological aspects of ALS biology, in particular oxidative stress and proteostasis, but also other plausible biological mechanisms with less research focus such as lipid metabolism (module 93) and neuron specific functions such as neurotransmission and synapse (module 128). Lipid metabolism dysfunctions as a driver of ALS pathology is currently much debated and aberrant lipid metabolism is proposed to underlie denervation of neuromuscular junctions mitochondrial dysfunction, excitotoxicity, impaired neuronal transport, cytoskeletal defects, inflammation and reduced neurotransmitter release [43]. The finding that one of our five ALS-core networks, one is linked to lipid metabolism is strengthening this view on the disease and may support the initiation of clinical trials investigating not only high fat diets, but also modulation of the balance between fatty acids and glucose oxidation [44] and modulation of sphingolipids [64].

The emphasis on proteostasis (protein degradation, modification and folding), which is part of all the presented networks and links genetic evidence (C9orf72, VCP, SQSTM, Dynactin, TBK) to altered chaperone functions (HSPS, crystallins, module 196) and autophagy (lysosomal degradation, modules 21 and 128) is also very interesting. The proteostasis network is a complex regulatory network that maintains protein homeostasis. It consists of several pathways that control protein biosynthesis, folding, trafficking, and degradation and responds to specific protein stress pathways such as the unfolded protein response (UPR), oxidative stress, inflammatory stress with regulation of autophagic lysosomal pathways, chaperones and heat shock response genes [59]. Therefore, dysregulation of this pathway can come therefore from many disturbances associated with ALS. Network 196 is associated with the proteasome pathway via HSPB1, which oligomerizes under oxidative stress, and TNF (regulation of an apoptotic response via DAXX) which links it to oxidative stress and inflammation. Network 21 contains FUS and TARDP, associating this network with RNA modifications and the autophagy process, which is another component of protein homeostasis [65]. Most of the genetic variations of ALS (C9orf72, TARDP and FUS) have recently been shown to be prone to aggregation, with possible a prion like mechanisms in ALS [66]. Apart from the loss of proper function of these proteins, these self-propagating aggregations are further impacting the proteasomal system [55]. Collapse of proper proteostasis due to failure to refold, degrade or effectively sequester and compartmentalize aggregation-prone, misfolded or potentially toxic proteins is detrimental to any cell type. Neuronal cells appear to be particularly vulnerable to disturbances in proteostasis most probably because they are long-lived, large post-mitotic cells that are not able to dilute out protein aggregates during cell divisions. In addition, the individual networks contain protein that are over expressed in particular cell types, such as neurobiology associated proteins (module 128) microtubular proteins (module 128), or c (module 196), which suggests that not only neurons suffer from disturbances in these systems but also muscular cells, with high crystallin expression functionally linked to microtubular and intermediate filament integrity [67–69] may directly be impaired.

There are a few genes that are part of the majority of core modules. In particular HDAC6 is found in 4 of the 5 Tier 1 networks, suggesting a central role in many biological pathways underlaying ALS biology. HADC6 plays a role in RNA metabolism, cytoskeletal dynamics and proteodynamics and its regulation in ALS is linked to FUS and TARDP [70]. Our networks suggest an overlap between these molecular pathways making it difficult to identify a single causative pathway. It is however remarkable that HDAC6, together with FUS and TARDP is part of the novel core module 21, which is almost exclusively made up of ALS-associated genes. While the biology of this network is only weakly classified, it suggests that mutations in all these genes are leading to a highly overlapping pathomechanism. Recently, pharmacological inhibition of HDAC6 has been shown to restore axonal transport defects *in vitro* and also ER to Golgi transport by increased acetylation of α-tubulin [71]. Similarly, SQSTM and HSPH1 are found in 3 of the 5 core network modules.

This analysis was performed on the network level and therefore it is not limited to the previously identified ALS-associated proteins. The network modules also contain novel proteins not previously associated with ALS and these proteins are candidates for being identified involved in ALS-related processes as well.

An investigation of the proteins identified through an updated text mining and database search revealed, that while none of the new proteins from ALSoD were found in the Tier 1 or Tier 2 disease modules, 11 proteins from HuGE Navigator and 11 proteins identified through text mining of the scientific literature were found in at least one of the Tier 1 or Tier 2 modules (S2 Table). This finding further strengthens our approach and supports a functional role of these new genes in ALS.

It is remarkable that most of the genes currently associated with ALS are found in the five Tier 1 disease modules. This suggests that there is a functional link between these mutated proteins, which leads to the common clinical phenotype of ALS, independent of the individual mutations. In this context network 21 is highly interesting, as it suggests a direct link between genes involved in RNA modifications and genes that are part of protein homeostasis, which is currently the most discussed mechanism underlying the pathogenesis of ALS.

TARDBP is a good example how deregulation of the proper function of a single protein is linking to many intracellular pathways. In line with its nuclear and cytoplasmic functions TDP-43 is pivotal in multiple cellular functions from RNA processing steps to misfolding and granule formation in the cytoplasm. Its pathological translocation to the cytoplasm on the one hand leads to a loss of nuclear function with dysregulated transcription, splicing, stabilization and RNA transport downstream leading to a dysregulation of a large number of dependent proteins. Its pathological presence in the cytoplasm on the other hand leads to a gain of function with formation of toxic aggregates (stress granules), an overload of the misfolded Protein response leading to a proteinopathy, which is present in 95% of all ALS cases [72]. Along these lines, this recent review puts the TARDP-linked proteinopathy in the center of a spectrum of neurodegenerative diseases [73].

It is also interesting that C9orf72, which is frequently mutated in ALS, is not part of network 21 or any of the other core networks. This could suggest that the gain of toxic function due to the repeats in the intronic part of the *C9orf72* gene resulting in the formation of Dipeptide Repeats (DPR) might be more prominent than the concomitant loss of physiological function of the C9orf72 protein in autophagy. In addition, this RNA repeats- and DPR-mediated toxic mechanism is likely to be part of another biological process than the one covered by network 21.

When interpreting the disease overlap of the modules (Fig 4), it is important to consider that the genes associated with each disease were found using text mining of the scientific literature, and that the diseases investigated are known to often be mentioned in ALS related abstracts (Table 3). In the case of this study, extra care must be taken since all 50 diseases we used for comparison, were defined as the top 50 diseases most often being co-mentioned with ALS. However, this will also be true for any study working with well-known associations such as FD, AD, PD, HD and MS.

Following this word of caution, the most striking observation is actually the lack of overlap with most of the top 50 diseases, as mentioned in the results section. For example, only a single Tier 2 module has an overlap with MS biology, while 12, including 3 of the 5 Tier1 have an overlap with AD. This indicates that the overlap matrix in Fig 4 is not just driven by a generic overlap in literature between ALS and the top 50 diseases, but it represents a clear trend towards the overlap with a specific subset of diseases.

Biologically, it is interesting that these core modules are so selectively represented in specific diseases. Oxidative stress shows a certain specificity to ALS (and associated FTD), while only module 128, with its many neurobiology associated genes shows a broader overlap. The modules often overlap with AD, either exclusively or together with other conditions, or they show a representation with muscular atrophy. This is an important hint on how general cellular processes may lead to specific diseases, and what pathways may be particularly vulnerable in both muscle and neurons such as the crystallin chaperone system (module 196). An interesting finding is the relatively high overlap of our core ALS networks in toxic encephalopathy. Toxic encephalopathy is a heterogeneous clinical disease of brain dysfunction caused by toxic substances of a wide variety [74]. MalaCards [75] reports several biological pathways (perinuclear transport, neuronal projection and membrane raft proteins) as targets of neurotoxic substances leading to clinical problem of encephalopathy. Many of these mechanisms are also central to ALS. This may support the speculation that pesticides and other environmental toxins can lead to ALS, which has been suggested as an explanation for the high incidence of ALS among military people and certain population groups as the Chamorro people of Guam through the exposure to the ß-methylamino-L-alanine (BMAA) found in fishes [76].

## Conclusions

In the case of complex diseases, discovering and describing the molecular systems responsible for the phenotype is extremely difficult, since a complex disease is not caused by a single gene, but is rather a perturbation of a biological system. Since the disease-causing genetic factors differ between individuals, it is crucial to understand how they are connected. Network analysis is an effective approach for investigating the functional interactions between molecules. Analyzing a comprehensive ALS dataset in context of protein-protein interactions allowed us to get a unique top-level understanding of ALS biology. By consolidating the networks into modules with known players in focus, we were able to extract a comprehensive and rich set of ALS modules.

When focusing on the five most significant modules (Tier 1), the represented biology is covering the main hypothesis around the pathogenesis of ALS, including oxidative stress, energy metabolism, proteasome dysfunctions and mRNA processing changes. Some of the modules are generic and shared with other neurological diseases. These involve the functional response to stress, be it oxidative or linked to protein- or energy metabolism. Many known ALS mutations lead to a dysregulation of the proper production of proteins, potentially starting at mRNA processing and resulting in a disturbed proteostasis, with a central role of TARDP. As neurons may be particularly sensitive to these failures, several networks are part of other neurodegenerative diseases (modules 128 and 21) and may be the molecular basis of a proteinopathy spectrum of diseases from dementias to muscular atrophies. Other networks, in particular the ones around SOD, are associated only with ALS (modules 83, 93 and 196). A follow-up study including recently identified ALS-associated proteins found that the networks we defined as “Core Modules” (Tier 1 and Tier 2) contained a significantly higher proportion of new ALS genes than expected.

## Supporting information

S1 Table ALS-gene list-new support. (XLSX)

S2 Table ALS protein list new2020. (XLSX)

S3 Table GO CLASS+SUPERCLASS. (XLSX)

S4 Table TM top50 table. (XLSX)

S1 File ALS disease modules. (DOCX)

S2 File Network artefacts. (CYS)

S1 Fig Network summary. Final networks.
(TIF)

## List of abbreviations

AD: Alzheimer’s Disease
ALS: Amytrophic Lateral Sclerosis
ALSoD: ALS online Database
AP-MS: Affinity Purified Mass Spectrometry
CNS: Central Nervous System
ER: Endoplasmatic Reticulum
fALS: Familial Amytrophic lateral sclerosis
FTD: Frontotemporal dementia
GWAS: Genome Wide Association Studies
HD: Huntigton’s Disease
KEGG: Kyoto Encyclopedia of Genes and Genomes: https://www.genome.jp/kegg/
LBD: Lewy Body Dementia
MND: Motor Neuron Disease
MAPs: Microtubule Associated Proteins
MS: Multiple Sclerosis
PD: Parkinson’s Disease
PPI: Protein-protein interaction
sALS: Sporadic Amytrophic lateral sclerosis
UniProt: A hub for protein Information: www.uniprot.org
