# Supplementary material for: A molecular view of amyotrophic lateral sclerosis through the lens of interaction network modules
Source: PLoS One. 2022 May 16;17(5):e0268159. doi: 10.1371/journal.pone.0268159 (PMC9109932; doi:10.1371/journal.pone.0268159)
Supplement: S1 File — (DOCX) [file pone.0268159.s005.docx]

# Title

A molecular view of amyotrophic lateral sclerosis through the lens of interaction network modules

# Authors

Klaus Højgaard Jensen^1¶^, Anna Katharina Stalder^2¶^, Rasmus Wernersson^1,3¶*^, Tim-Christoph Roloff-Handschin^2^, Daniel Hvidberg Hansen^1^ and Peter MA Groenen^4*^

^1^Intomics A/S, Copenhagen, Denmark

^2^University Hospital Basel, Switzerland

^3^DTU Health Technology, Technical University of Denmark, Lyngby, Denmark

^4^Idorsia Pharmaceuticals Ltd. Allschwil Switzerland

^¶^These authors contributed equally to this work.

^*^Corresponding authors

**Email:** [rwe@intomics.com](mailto:rwe@intomics.com) (RW), [peter.groenen@idorsia.com](mailto:peter.groenen@idorsia.com) (PM)

**All email addresses:** KHJ: [khj@intomics.com](mailto:khj@intomics.com), AKS: [annakatherina.stalder@usb.ch](mailto:annakatherina.stalder@usb.ch), RWE: [rwe@intomics.com](mailto:rwe@intomics.com), TCRH: [tim.roloff@usb.ch](mailto:tim.roloff@usb.ch), DHH: [dah@intomics.com](mailto:dah@intomics.com), PG: [peter.groenen@idorsia.com](mailto:peter.groenen@idorsia.com)

**ORCIDs:** KHJ: [0000-0003-3234-6068](https://orcid.org/0000-0003-3234-6068)**,** AKS: [0000-0002-8525-758x](https://orcid.org/0000-0002-8525-758X), RWE: [0000-0003-4417-9842](https://orcid.org/0000-0003-4417-9842), TCRH: [0000-0003-3435-4723](https://orcid.org/0000-0003-3435-4723), DHH: [0000-0003-3285-605X](https://orcid.org/0000-0003-3285-605X), PG: [0000-0002-8172-1752](https://orcid.org/0000-0002-8172-1752)

**Keywords:**

Amyotrophic lateral sclerosis, ALS, neurodegenerative disease, protein-interaction networks, PPI, data integration, Network Biology, molecular disease mechanism

# SUPPLEMENTARY MATERIALS

## Review of node degree bias in protein-protein interaction networks

As mentioned in the main text, it is important to be aware of underlying biases in the interaction data when working with protein-protein interaction (PPI) networks, as false conclusions may otherwise be reached when conducting the downstream analysis.

An important factor to consider, is that certain proteins have the problem of being associated with an inflated number of interaction partners. As a result, they may connect otherwise unrelated parts of the global network, and purely due to their "over-connectedness" appear to be interacting with key genes/proteins in a particular biological system under investigation (in this case ALS). Two major factors should be taken into consideration: 1) Experimental artifacts and 2) Study bias.

Important experimental artifacts to consider in this type of data are randomly distributed noise and unspecific binding of proteins. As with all other experimental data, the determination of protein-protein interactions is affected by experimental noise (false positives and false negatives). The degree of the noise depends on the nature of the experimental system (e.g. yeast two-hybrid (Y2H) and affinity-purification mass spectrometry (AP-MS)) as well as the nature of the study [1]. Small-scale studies with few interaction partners are often considered less noisy compared to large scale studies, where less attention to detail may be given to individual interactions. inBio Map [2] handles the uncertainty in the underlying experimental data, by assigning a confidence score to each interaction, which includes a measurement of the number of independent studies supporting the interaction as well as the nature of the study (small-scale vs. large-scale) - see Li et al [2], supplement for all details. This allows us to rank all interactions based on experimental support, and only use the high-confidence subset of interactions. This precaution addresses the part of the problem, that is due to randomly distributed noise in the data, which can indeed be substantial when integrating 40,000+ experimental studies.

However, in AP-MS experiments certain proteins and protein families tend to be co-purified with other proteins under investigation due to unspecific binding. This can even be unspecific binding to the affinity-purification column itself. The CRAPome project [3] has investigated this in great detail, and across 300+ negative controls (AP-MS experiments run without the "bait" protein present) more than 100 proteins were seen in more than 50% of the experiments. Worst offenders are heat-shock proteins (HSPs) with more than 90% presence, tubulins/actins with more that 80% presence, and generally highly expressed proteins such as histones and elongation factors with more that 50% presence. To complicate the matters further, certain post-translational modifications (PTM) often show up as false positives in mass-spectrometry based studies, for example when a (small) protein or peptide is covalently attached to another protein. This will appear as if the attached protein/peptide is interacting with the other protein. Common examples of this includes UBB, UBC, UBA52, RPS27A, SUMO1, SUMO2, SUMO3 and SUMO4, which are often covalently attached to other proteins in an unspecific manner. Unspecific should in this context be understood in the following way: Protein-protein interactions are most often between proteins that work in the same biology, whereas peptides flagging other proteins for degradation can be covalently linked to a broad range of proteins not likely to be otherwise related. It should be noted that UBB and UBC are also detected in more than 50% of the negative controls in the CRAPome study. Ideally, AP-MS experiments should correct for unspecific binding before reporting the results, but this is not always the case [3], and it may be difficult to completely eliminate the false positive signal.

Finally, a fundamentally different issue that needs to be considered is study bias. While some protein-protein interaction experiments lend themselves well to genome-wide studies (e.g. Y2H screens), the AP-MS class of experiments are fundamentally built to identify interaction partners for sets of proteins of particular interest. In essence, the study bias in the proteome (with much of the proteome being understudied) is carried over into study of the interactome.

Good examples of the issues of overrepresentation of highly connected proteins in the PPI resource is the heat-shock protein HS90A and many of the ubiquitin-like modifiers which are some of the most studied proteins in literature (top 10 proteins with most connections in our PPI network are shown in Supplementary Table S 5 (below). This is important, since network analysis based studies that are built around the concept of the "*centrality-lethality rule*" [4] where the node degree (number of interaction partners) is important, are likely to pick up exactly this class of over-connected proteins, unless a node-degree normalization scheme is used.

**Table S 5**

Top 10 most highly connection proteins in the global PPI network.

| **UniProt ID** | **# of interactions** | **UniProt AC** | **Description** |
| --- | --- | --- | --- |
| SUMO1_HUMAN | 778 | P63165 | Small ubiquitin-related modifier 1 |
| SUMO2_HUMAN | 633 | P61956 | Small ubiquitin-related modifier 2 |
| P53_HUMAN | 587 | P04637 | Cellular tumor antigen p53 |
| RS27A_HUMAN | 556 | P62979 | Ubiquitin-40S ribosomal protein S27a |
| RL40_HUMAN | 532 | P62987 | Ubiquitin-60S ribosomal protein L40 |
| HDAC1_HUMAN | 499 | Q13547 | Histone deacetylase 1 |
| GRB2_HUMAN | 484 | P62993 | Growth factor receptor-bound protein 2 |
| UBC_HUMAN | 454 | P0CG48 | Polyubiquitin-C |
| SUMO3_HUMAN | 447 | P55854 | Small ubiquitin-related modifier 3 |
| HS90A_HUMAN | 447 | P07900 | Heat shock protein HSP 90-alpha |

# References

1. von Mering C, Krause R, Snel B, Cornell M, Oliver SG, Fields S, et al. Comparative assessment of large-scale data sets of protein–protein interactions. Nature. 2002;417:399–403. doi:10.1038/nature750.

2. Li T, Wernersson R, Hansen RB, Horn H, Mercer J, Slodkowicz G, et al. A scored human protein–protein interaction network to catalyze genomic interpretation. Nat Methods. 2017;14:61–4. doi:10.1038/nmeth.4083.

3. Mellacheruvu D, Wright Z, Couzens AL, Lambert J-P, St-Denis NA, Li T, et al. The CRAPome: a contaminant repository for affinity purification–mass spectrometry data. Nat Methods. 2013;10:730–6. doi:10.1038/nmeth.2557.

4. Jeong H, Mason SP, Barabási A-L, Oltvai ZN. Lethality and centrality in protein networks. Nature. 2001;411:41–2. doi:10.1038/35075138.
