# Supplementary figures and images for: A molecular view of amyotrophic lateral sclerosis through the lens of interaction network modules
Source: PLoS One. 2022 May 16;17(5):e0268159. doi: 10.1371/journal.pone.0268159 (PMC9109932; doi:10.1371/journal.pone.0268159)

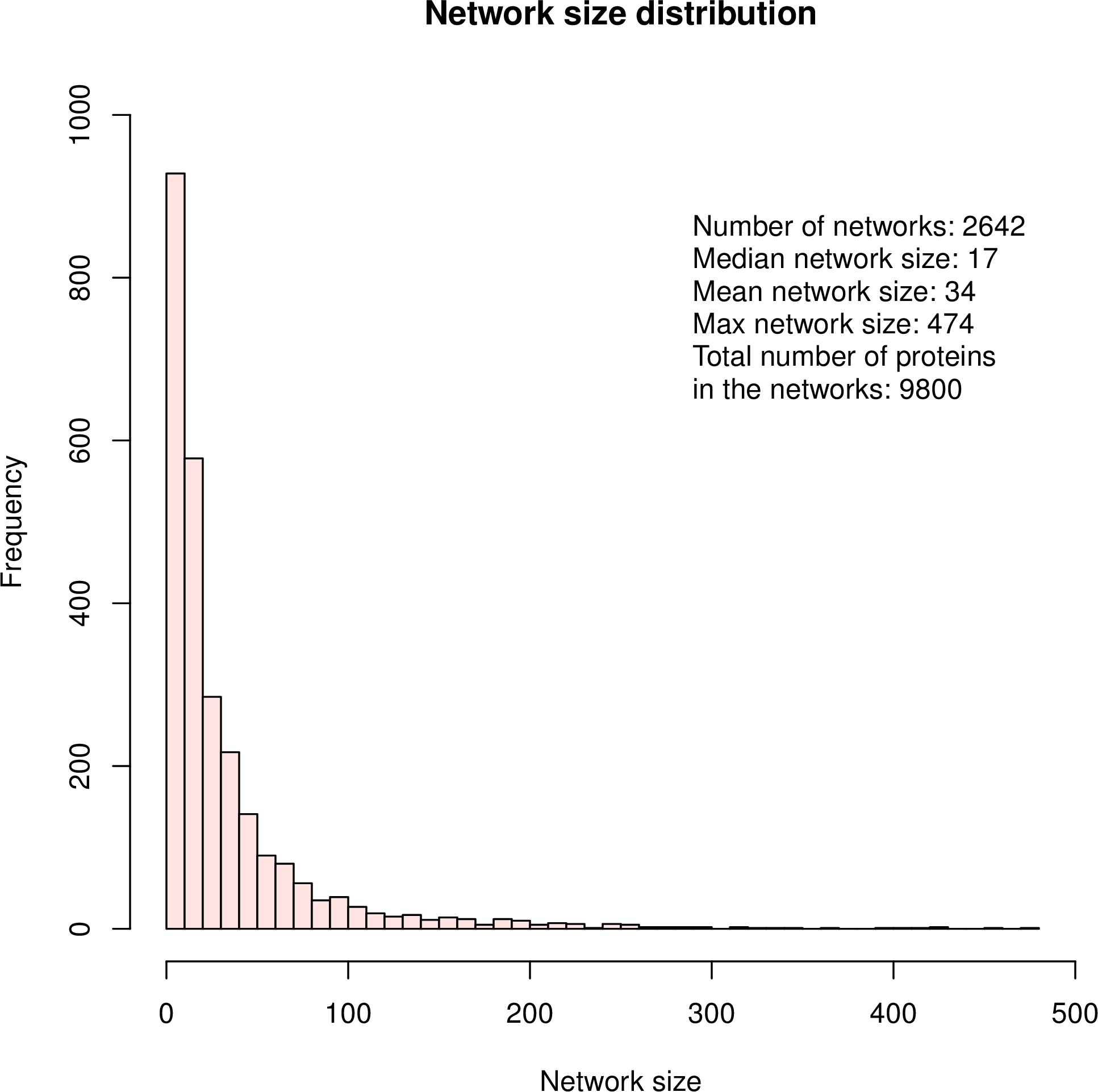

Supplement: S1 Fig — Final networks. (TIF) [file pone.0268159.s007.tif]
